# Two p53 tetramers bind one consensus DNA response element

**DOI:** 10.1093/nar/gkw215

**Published:** 2016-03-31

**Authors:** Sinéad Kearns, Rudi Lurz, Elena V. Orlova, Andrei L. Okorokov

**Affiliations:** 1 Institute for Structural and Molecular Biology, School of Biological Sciences, Birkbeck College, London WC1E 7HX, UK; 2 Wolfson Institute for Biomedical Research, Division of Medicine, University College London, London WC1E 6BT, UK; 3 Max Planck Institute for Molecular Genetics, Ihnestrasse, Berlin 14195, Germany

## Abstract

p53 tumor suppressor is a transcription factor that controls cell cycle and genetic integrity. In response to genotoxic stress p53 activates DNA repair, cell cycle arrest, apoptosis or senescence, which are initiated via p53 binding to its specific DNA response elements (RE). The consensus p53 DNA RE consists of two decameric palindromic half-site sequences. Crystallographic studies have demonstrated that two isolated p53 DNA-binding core domains interact with one half-site of the p53 DNA REs suggesting that one p53 tetramer is bound to one RE. However, our recent 3D cryo-EM studies showed that the full-length p53 tetramer is bound to only one half-site of RE.

Here, we have used biochemical and electron microscopy (EM) methods to analyze DNA-binding of human and murine p53 tetramers to various p53 DNA REs. Our new results demonstrate that two p53 tetramers can interact sequence-specifically with one DNA RE at the same time. In particular, the EM structural analysis revealed that two p53 tetramers bind one DNA RE simultaneously with DNA positioned between them. These results demonstrate a mode different from that assumed previously for the p53-DNA interaction and suggest important biological implications on p53 activity as a transcriptional regulator of cellular response to stress.

## INTRODUCTION

The p53 transcription factor, encoded by the *TP53* gene, is a major tumor suppressor that controls genetic integrity and cell proliferation (1). In response to various forms of stress, p53 is activated and accumulates in the nucleus, where it regulates the transcription of numerous target genes via interaction with its specific DNA response elements (RE), components of transcription machinery and chromatin remodeling factors (2–4). Depending on the type and amount of stress, and the type of tissue, the p53-dependent response leads to DNA repair, cell cycle arrest, metabolic reprogramming, apoptosis or senescence, thus preventing the development of cancer (5). Mutations in the *TP53* gene are associated with more than a half of all forms of human malignancies (6–8).

Human p53 protein is a polypeptide of 393 amino acid residues in length that forms tetramers in solution, in a dimer-of-dimers manner (9–15). p53 consists of five domains: transcription activation domain (residues 1–67), a proline-rich region (residues 67–98), a central core domain (residues 98–303), a nuclear localization signal-containing region (303–323), the oligomerization domain (residues 323–363) and the C-terminal basic domain (residues 363–393) (16,17). Unlike other transcription factors, p53 has two DNA-binding domains. One is the core domain responsible for binding to sequence-specific DNA REs located near promoters of the p53 target genes (18–21). Most of the cancer-associated missense mutations occur in the sequence-specific DNA-binding core domain, where mutations either disrupt protein–DNA interactions directly or alter its overall conformation (22). The second is the C-terminal domain of p53 that forms stable complexes with non-specific DNA, including mismatched DNA, double-strand breaks and single-stranded DNA (23,24). It has been proposed that the C-terminus of p53 also provides additional anchorage to specific DNA sites via non-specific flanking interactions, thus stabilizing the whole complex (25–30). The latter was supported by electron microscopy (EM) structures of the full length p53 and p53-DNA complex where the C-terminal domains were localized in the close proximity to DNA (31,32).

A p53 consensus DNA RE is composed of a tandem of two decameric palindromic sequences (half-sites) 5′-RRRCWWGYYY-3′, where R = purine, Y = pyrimidine and W is either A or T. There is a variability in composition of p53 REs, thus two half-sites can be separated by a spacer DNA, typically 0–13 bp in length and many p53 DNA REs have varying numbers of half-sites (19,20,22,33–37). Early biochemical studies in conjunction with electron microscopy showed that p53 binds DNA REs as a tetramer, with both dimers of each tetramer thought to be engaged in the binding (9–14,38). High molecular order complexes were also reported where multiples of p53 tetramers were bound to DNA REs presumably, as explained at that time, via tetramer to tetramer interactions (12).

The mechanism of DNA recognition by p53 was proposed on the basis of the crystallographic structure of the isolated p53 core domain bound to a specific DNA target (22). This finding was corroborated by more recent structures of the p53 isolated core domains in complex with the half-site and full-size RE DNAs (39–42). Based on these data a model of p53-DNA complex has been suggested where all four core domains of the p53 tetramer bind the full RE sequence in a fashion where the DNA is ‘wrapped’ up by p53 protein (22,39–44). However, it is hard to reconcile this model with DNA-binding in a chromatin context, where DNA is packed in nucleosomes. This model has been challenged by cryo-EM studies of the full length p53. A recent structure of the full-length murine p53 tetramer indicated that the p53 active complex is formed by a dimer of dimers where the monomers interact via their juxtaposed amino-terminal (N) and carboxy-terminal (C) domains to form N/C nodes (31).

The structure of the full-length p53-DNA complex showed that each of the p53 dimers contribute one core domain to form a complex with only one half-site of p53 DNA RE. This type of interaction suggests that a pair of core domains is located on one side of the tetrameric complex, while the other pair is on the opposite side, thus making the binding of the four core domains from the same p53 tetramer to the one 20 bp-long response element unlikely. This configuration of a p53 tetramer is further supported by the subsequent EM analysis of the murine full-length p53 tetramer in complex with specific DNA RE (32). The arrangement of core domains engaged in DNA-binding was identical to that described by crystallographic studies (32,40,41). The EM structures suggest that while one p53 tetramer is bound to one half-site of p53 RE, the other half-site remains unoccupied. This model of interactions raises questions about a role of the second half-site in DNA RE and whether it will be possible for the second p53 tetramer to bind the same DNA RE. Such a possibility would only be viable when two p53 tetramers bind p53 RE from the opposite sides of DNA, one tetramer per half-site using DNA RE sequence.

To test this possibility we used biochemical and EM approaches. Modified gel-shift experiments of p53-DNA complexes cross-linked with glutaraldehyde were used to demonstrate that two p53 tetramers can bind one consensus DNA RE leading to a p53 double tetramer-DNA complex. Our experiments revealed the same results for both murine and human p53 proteins, suggesting a conserved mechanism. Furthermore, the p53 double tetramer-DNA complexes were extracted from native protein gels by a modified gel-to-grid technique and visualized using negative stain electron microscopy. Image analysis confirmed that two p53 tetramers are bound to one DNA RE simultaneously with DNA located in between two tetramers. Our results indicate a mechanism of joint p53 tetramer interaction with DNA RE that can facilitate looping of distal p53 DNA binding sites and provide a dose-dependent response to stress, thus having important biological implications on p53 transcriptional activity in regulating cellular fate.

## MATERIALS AND METHODS

### Recombinant p53 and p53-DNA complex preparation

Recombinant murine and human p53 proteins were expressed in the baculoviral system using Sf9 cells, purified and tested for homogeneity as described previously (31). Freshly purified proteins were used for DNA-binding assays using various double-stranded DNA (dsDNA) targets, which were prepared by annealing their complementary DNA strands (Table 1).

**Table 1.** Oligonucleotide DNA targets used in p53 DNA-binding experiments. Arrows (→ or ←) represent p53 RE quarter sites. (→← represent p53 RE half-sites). Corresponding forward (fwd) and the complementary reverse (rev) oligonucleotides were annealed to each other produce the double-stranded DNA targets. DNA targets #1-9 were as reported in (13). DNA sequences from natural promoters were adapted from *p21* (19), *gadd45* (50), MCK (51), *RGC* (18), *Mdm2* (52) and *bax* (54)

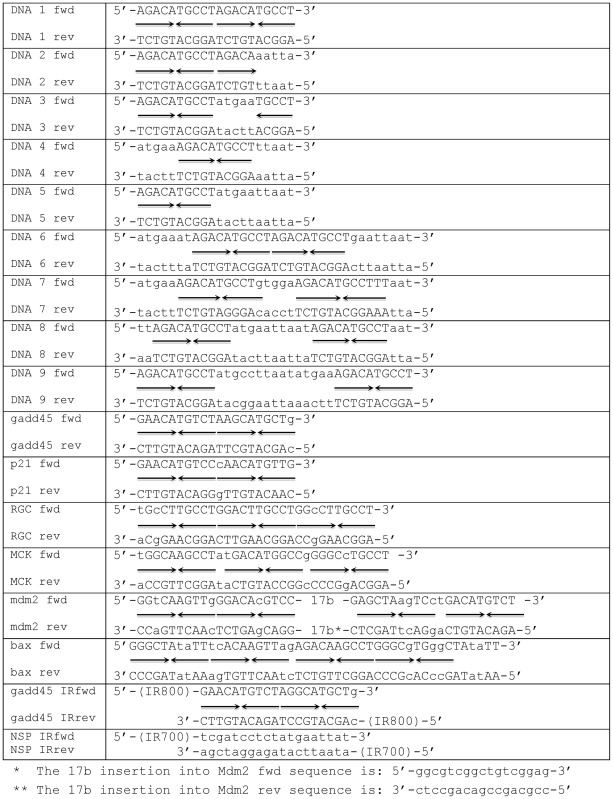	

p53-DNA complexes were formed by mixing p53 with a DNA target of choice and the final volume of DNA-binding mixtures was 50 μl, of which the DNA template was typically 5 μl (maximum of 250 ng per reaction) and the remaining volume was made of the p53 protein with maximum of 2.5 μg of p53 per reaction, and the reaction buffer based on Tris (25 mM Tris-HCl, pH 7.5, 2 mM DTT, 100 mM NaCl, 1 mM ADP, 5 mM of MgCl_2_) or HEPES (20 mM HEPES, pH 7.5, 2 mM DTT, 100 mM NaCl, 10 mM ADP, 5 mM of MgCl_2_) where indicated. When HEPEs was used as a buffer for crosslinking and DNA-binding reactions the recombinant p53 proteins were also eluted in HEPES-based buffer. Different ratios of DNA and protein were used at the initial stages of this work to determine the best DNA-binding conditions as described in the results. p53-DNA reaction mixtures were prepared in 1.5 ml Eppendorf tubes, incubated on ice for 5 min, then for 15 min in a thermostated mixer at 25°C with 450 rpm shaking after every 20 s. This was followed by the addition of 0.025% glutaraldehyde (GA), which was freshly prepared from 25% glutaraldehyde (Sigma) using ultra-pure water. Typically a volume of 5 μl 0.25% GA was added to 50 μl reaction mixtures to make final concentration of GA 0.025% and incubated at 25°C for another 20 min. Reaction samples were then loaded onto either denaturing or native protein gels using the appropriate sample-loading buffer. For initial time-course experiments, cross-linking was initiated by the addition of various amounts of GA as described in the results.

### Electro-mobility shift assays (EMSA)

Twenty bp long DNA specific and non-specific targets were prepared by annealing their corresponding oligonucleotides. Both strands of each target were 5′-labeled by an IR-dye, IR800 for specific (gadd45) DNA and IR700 for non-specific DNA, resulting in green and red signals when detected by the LI-COR Odyssey CLx system. p53 protein–DNA complexes were prepared and cross-linked with 0.025% GA as described above and loaded onto either 4–12% MOPS SDS gel (Invitrogen) or Native PAGE Novex 4–16% Bis-Tris gel (Invitrogen). The loading buffers were DTT-containing 3x loading buffer (BioRad) for denaturing PAGE and 5% glycerol and 0.01% Ponceau S loading buffer for the native gels (Invitrogen). Typically a 20 μl sample of each reaction was loaded in each well. 1x MOPS SDS buffer (Invitrogen) was used for the denaturing PAGE. Anode (25 mM imidazole. pH 7) and cathode (50 mM tricine, 7.5mM imidazole, 0.002% Coomassie blue-G250. pH 7) buffers were used for the Native PAGE.

Denaturing electrophoresis was carried out at 4°C, or on ice for approximately 3.5 h at 150 V, resulting in 50 kDa Mw protein marker migrating approximately three-quarters of the gel length. Native electrophoresis was also carried at 4°C, for ∼3.5 h at 150 V, 8–10 mA. The cathode buffer for the latter was replaced when the current dropped to 2 mA, approximately every hour and a half. Gels were analyzed by either coomassie or silver staining for protein content and by scanning with LI-COR Odyssey system to detect IR-dye labelled DNA. Gels containing IR-labelled DNA were analyzed and quantified using ImageStudioLite software (LI-COR). For immunoanalysis (Western blotting) gels were transferred onto nitrocellulose C (Amersham) using semidry BioRad apparatus at 15 V for 1 h and CAPS transfer buffer (10 mM CAPS, 20% Methanol, 0.02% SDS), blocked with 5% fat-free milk, probed with PAb 240 as primary antibody (in 5% fat-free milk) overnight at 4°C and rabbit anti-mouse horseradish peroxidase (HRP) -conjugated secondary antibody (Dako) for 1 h at room temperature, washed and developed using Pierce^TM^ enhanced chemiluminescence (ECL) Western blotting substrate.

### Gel-to-grid transfer

p53-DNA complexes were prepared and separated as described above and resolved on Native (Blue) PAGE Novex 4–16% Bis-Tris gels (Invitrogen). The position of the p53-DNA complexes in the gel were determined by detecting the IR-dye labelled DNA targets used in the p53-DNA complexes by the Odyssey Imaging System (LI-COR). Only one side of the plastic gel cast was removed before the scanning procedure, so that the positions of the complexes could be marked on the remaining side of the cast using a permanent marker, which was visible under IR light and thus could be re-verified by a quick scan. Zones of the gel containing p53-DNA complexes of interest were excised by using a scalpel. The glow-discharged carbon-coated copper grid was placed carbon side facing up onto a 2 mm thick glass plate (BioRad Mini-Protean II system), an excised piece of gel was positioned onto the grid and a 5 μl droplet of electrophoresis buffer was placed on top of the gel slab to prevent extra drying. The blotting assembly sandwich was finalized by placing another identical glass plate on top. A 300 g weight (a conical glass flask filled to the weight with water) was positioned on top of the glass plate sandwich and left for 2 min to accelerate the protein–DNA particles diffusing from gel onto the grid (Supplementary Figure S8A). After 2 min the grid was taken out of the glass plate sandwich and stained with uranyl acetate as described below. Typically 3 grids and 3 gel slabs were blotted simultaneously in one assembly allowing to process about 12–16 slabs cut from one 15-well gel in about 20 min.

### Electron microscopy

#### Rotary shadowing

The 0.025% GA cross-linked p53-DNA complexes were allowed to adsorb to freshly cleaved mica for 2 min. They were stained with 2% uranyl acetate for 2 min followed by three washes in water. Air-dried mica was rotary-shadowed with Pt-Ir alloy at an angle of 4–7° followed by carbon evaporation in an Edwards Coating System (E306A) (45). Grids were examined in a FEI CM 100 EM; images were taken with 1k x 1k TVIPS F114 slow-scan CCD camera at x11 500 nominal EM magnification.

#### Negative staining

Samples were applied to carbon-coated copper grids (square 400 mesh copper grids, 3 mm diameter, Agar Scientific) using the gel-to-grid transfer method. The grids were then washed with 3 μl buffer (100 mM NaCl, 25 mM Tris-HCl pH 7.5), blotted and stained with 3 μl uranyl acetate (2%, pH 4.5) for 2 min. The grids were blotted once more, dried and analyzed under the microscope. A preliminarily examination was carried out on a Tecnai T12 electron microscope at 120 kV to assess grid quality and sample and stain distribution followed by the data collection using Tecnai F20 FEG electron microscope. The images were recorded under low electron-dose conditions using a Gatan Ultrascan 4000, 4k × 4k CCD camera at a nominal magnification of x50 000 (pixel size of 3.6 Å/pixel). A range of defocus values varying from 1.5 to 2.5 μm was used for data collection.

### Image processing

Particles from the gel-to-grid transfer images were manually picked using the EMAN Boxer software package (46). The contrast transfer function of the microscope was determined using the CTFFIND3 program and then corrected by phase flipping (47). The images were then normalized to the same mean grey values and standard deviation followed by band-pass filtering to remove uneven background and high frequency noise (with low- and high-resolution cut-offs of 90 Å and 12 Å, respectively). Alignment and classification were performed using the IMAGIC-5 software package (48).

Surface rendering was performed using a threshold level of ∼2 standard deviations (2σ) in the maps corresponding to ∼100% of the expected mass of the complex. Figures were generated using Chimera (49).

## RESULTS

### p53 forms high molecular order complexes on its specific DNA RE

**Figure 1.**
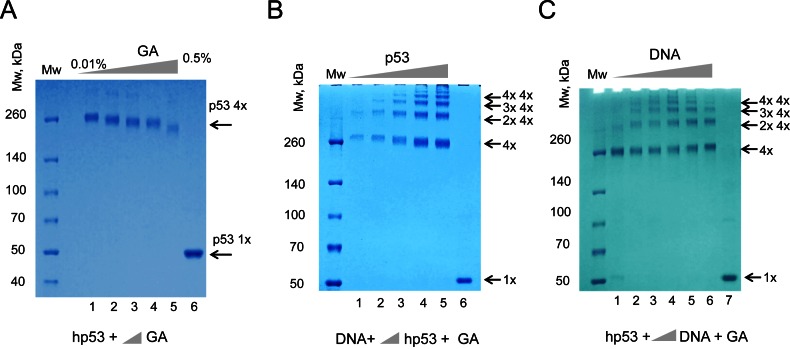
p53 tetramers form high molecular order complexes on DNA RE. (**A**) Equal amounts of the recombinant human p53 (2.5 μg) were cross-linked with increasing concentrations of glutaraldehyde (GA). Lane 1 – 0.01%; Lane 2 – 0.025%; Lane 3 – 0.05%; Lane 4 – 0.1% and Lane 5 – 0.5%) and products of reactions were separated using 4–12% MOPS SDS PAGE. Lane 6 – control, no GA was used. (**B** and **C**) Complexes of the recombinant human p53 with dsDNA (DNA#1, 20 bp) were cross-linked with GA (0.025%) and analyzed by 4–12% MOPS SDS PAGE stained with coomassie G-250. Increasing amounts of p53 (0.25, 0.5, 1.25, 2.5 and 5 μg) were used in (B) and increasing amounts of DNA targets with 2 μg of p53 were used in (C). Products of crosslinking resolved in a ladder-like pattern of high molecular order complexes, molecular masses of which corresponded to one, two, three, four and higher amount of p53 tetramer per complex. Increase of DNA or protein amounts did not change the stoichiometry of complexes. Lane 6 (B) and Lane 7 (C) – controls with no GA added to p53.

To confirm that the recombinant p53 used in our experiments was a tetramer we used chemical cross-linking with glutaraldehyde (final concentration from 0.01% to 0.5%) followed by denaturing protein SDS-PAGE (Figure 1A). These experiments confirmed that even at low amount of glutaraldehyde recombinant human p53 was a tetramer in solution, migrating as a complex of about 260 KDa molecular mass range which was in agreement with all published reports that p53 is tetrameric even at low nM concentrations (10–13,15). Increasing amounts of glutaraldehyde (up to 0.5%) caused visible change in migration due to excessive crosslinking Figure 1A. A final concentration of 0.025% of glutaraldehyde was chosen as optimal for all the following experiments.

To test a hypothesis that p53 forms double tetramers on a consensus sequence-specific 20 bp DNA target, (DNA1, Table 1) recombinant human p53 was incubated with DNA RE, cross-linked with glutaraldehyde and analyzed on protein SDS-PAGE. The cross-linked p53-DNA complexes were resolved in a ladder-like pattern which represented a range of high molecular order complexes of p53 with DNA, molecular masses of which corresponded to one, two, three, four and higher numbers of the p53 tetramers per complex with increments of ∼200–250 kDa. This result indicated that in solution p53 and its specific DNA target form complexes that comprise multiple copies of p53 tetramers (Figure 1B and C). This type of DNA-binding by multiple p53 tetramers was evident in all cases with different ratios of p53 tetramers per DNA (from 1:1 to 4:1). The ratio between single, double and triple p53 tetramers bound to DNA remained proportional when different amount of p53 were used in reactions (Figure 1B). Analogous results were obtained for murine p53, indicating that protein sequence differences between these two homologues do not affect their DNA-binding behavior (Supplementary Figure S1).

### DNA-binding of multiple p53 tetramers to DNA RE is sequence specific

**Figure 2.**
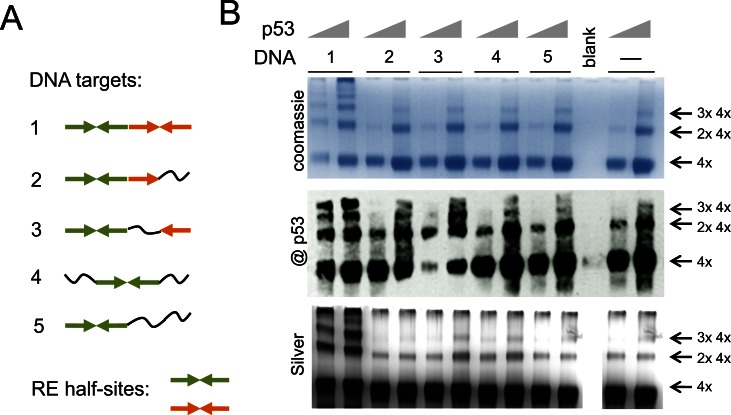
Multiple binding of p53 tetramers to DNA RE is sequence specific. p53 forms high molecular order complexes with DNA and this process depends on the amount of half-sites in DNA RE. (**A**) DNA targets composed of different amount half-sites and quarter sites. Quarter site sequence RRRCW depicted as → and quarter site sequence WGYYY depicted as ←, where R = purine, Y = pyrimidine and W is either A or T. The complete half-site is →←. Different colors are used to show two adjacent half-sites. **∼** – non-specific DNA sequence. (**B**) Recombinant p53 (murine, 1.25 and 2.5 μg) can only efficiently form high order molecular complexes with DNA targets that have both half-sites (DNA #1). p53 forms double tetramers in solution but it is not stimulated any further by DNA targets #2–5, control – last two lanes, no DNA. Immunoblotting was done with Pab240.

We next examined whether this multiple DNA-binding by p53 tetramers takes places on DNA targets that have less and/or degenerated half-sites sequences than the consensus p53 DNA RE. We used DNA targets of the same length (20 bp) with the internal sequences of the decameric half-sites being reorganized to disrupt internal palindromic repeats (Figure 2A, DNA targets 1–5). The formation of complexes with different dsDNA targets has been analyzed at different concentrations of p53. The p53-DNA complexes were formed as described above and fixed with glutaraldehyde (0.025%). SDS-PAGE analysis showed that the DNA-binding by multiple p53 tetramers depends on the number of half-sites in the DNA RE. p53 could only efficiently form high order molecular complexes with DNA targets that have two half-sites (DNA1) (Figure 2B). The DNA targets of types 2–5 did not stimulate DNA-binding of multiple p53 tetramers when compared to those in the absence of DNA (Figure 2B, control (−) − no DNA). The complexes have been clearly identified by both coomassie and silver staining and the p53 nature of these complexes was confirmed by immunoblotting (Figure 2).

To see whether the length of DNA targets and/or conditions of cross-linking reactions could have an effect on the observed p53-DNA complex formation we also tested longer DNA targets which had extra 23 bp-long flanks on both sides of the target sequences (Supplementary Table S1) and reactions were repeated using HEPES buffer instead of Tris (Supplementary Figure S2). When tested mp53 showed results similar to those observed in Figure 1 and Supplementary Figure S1 indicating that neither change of the length of DNA nor buffer conditions were affecting the multiple p53 tetramers binding to DNA. Furthermore the truncated version of mp53 lacking the last 30 amino acid residues (mp53Δ30) showed behavior similar to that of the full-length mp53 protein (Supplementary Figure S2B).

**Figure 3.**
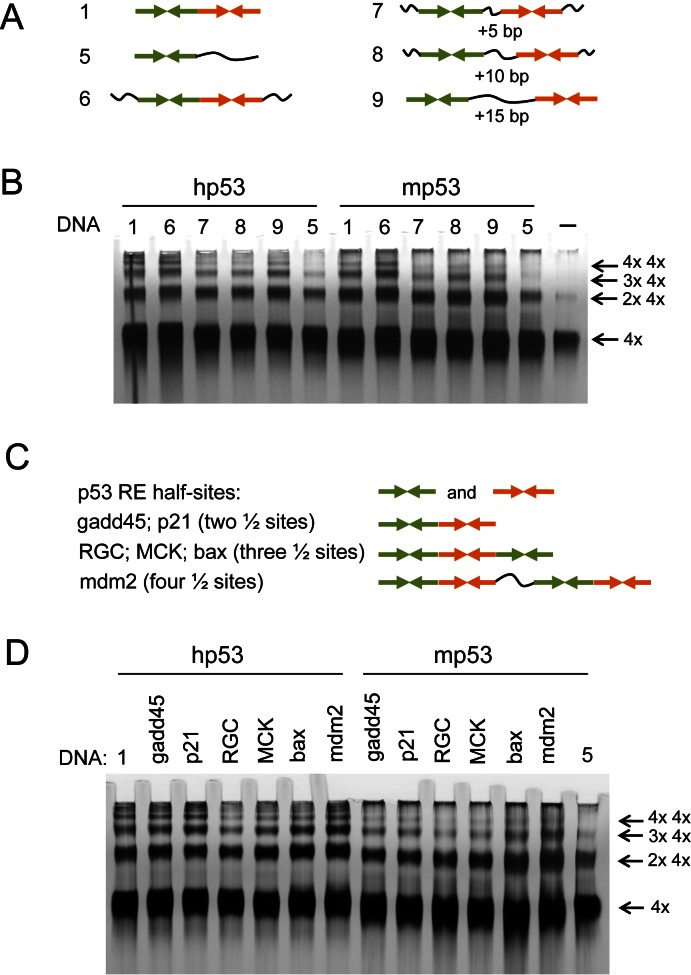
DNA-binding of multiple p53 tetramers to the natural RE sequences. (**A**) DNA targets with different spacer length between the RE half-sites were used to test their ability to promote high molecular order p53-DNA complexes. The half-sites depicted as →←. **∼** - non-specific DNA sequence. (**B**) Both human and murine p53 form multiple complexes on DNA targets with different spacer length between the half-sites (DNA targets # 6–9). DNA targets without any spacer (DNA targets #1 & 6) appear to be more efficient in stimulating high order complexes. (**C**) RE DNA targets derived from natural promoter sequences were used to test their ability to promote high molecular order p53-DNA complexes. Gadd45 and p21 sequences have two, RGC, MCK and bax with 3 and mdm2 have four half-sites respectively. The half-sites are depicted as →←. (**D**) Human p53 appears to be more efficient in multiple binding of DNA RE targets than its murine homologue.

Whilst it became clear that the DNA-binding by multiple p53 tetramers was stimulated by the sequence-specific interaction with the DNA RE half-sites it was also essential to examine the role of the spacer length between RE half-sites. We tested the DNA-binding of both human and murine p53 to DNA targets with the spacer length varying from 0 to 15 bp between the RE half-sites (Figure 3A). The most efficient DNA-binding targets to form complexes larger than eight p53 tetramers bound to DNA were those with two half-sites adjacent to each other (Figure 3B). Thus, DNA targets 1 and 6, both of which had no spacer between the half-sites of RE, gave identical results for both human and murine p53 proteins and efficiently promoted formation of complexes as large as two, three and four p53 tetramers bound to DNA. DNA targets having a spacer length of 5 bp (DNA7), 10 bp (DNA8) and 15 bp (DNA9) were less efficient in promoting p53-DNA high molecular order complexes, with the DNA9 target with the 15 bp linker being most efficient of them (Figure 3B).

Another factor that may affect the DNA-binding for human and murine p53 protein tetramers was the origin of DNA RE sequences. We analyzed six DNA targets derived from the natural promoters where RE sequences had different numbers of half-site decamers: RE sequences from *gadd45* and *p21* genes have two, REs from *RGC, MCK* and *bax* have three, and RE from *mdm2* have four half-sites (Table 1, Figure 3C). All these targets promoted DNA-binding of multiple p53 tetramers (Figure 3D). The most efficient DNA targets for murine p53 were derived from *bax* and *mdm2* (Table 1, Figure 3C and D). At the same time DNA targets derived from *RGC* and *MCK* RE sequences were less efficient in promoting p53-DNA complexes larger than 8 and 12 p53 tetramers. Interestingly, the human p53 showed more of the multiple tetramers DNA-binding than its murine homologue on all natural sequences used, that is forming more complexes with three and four p53 tetramers bound to DNA. The observed difference could potentially be explained by the source of the DNA sequences used, all but one (mdm2) were from human genome.

To further test whether the spacer length shorter than 5 bp may have an effect on DNA-binding by multiple p53 tetramers we used DNA targets 21 and 31–37 (Supplementary Table S1) with spacer length varying from 0 to 7 bp between the RE half-sites (Supplementary Figure S3A). Both human and murine p53 showed similar results in that the DNA-binding by multiple p53 tetramers was only at its most efficient when spacer length was 0 or 1 bp (Supplementary Figure S3). This was also consistent with the data obtained when DNA targets derived from TIGAR promoter p53 RE were used. TIGAR p53 RE has almost perfect consensus sequence but 2 bp spacer in between of its two half-sites (54). When the spacer was removed the binding of multiple p53 tetramers was increased for both human and murine p53 (Supplementary Figure S3).

### The amounts of p53 and DNA are proportional in all complexes

**Figure 4.**
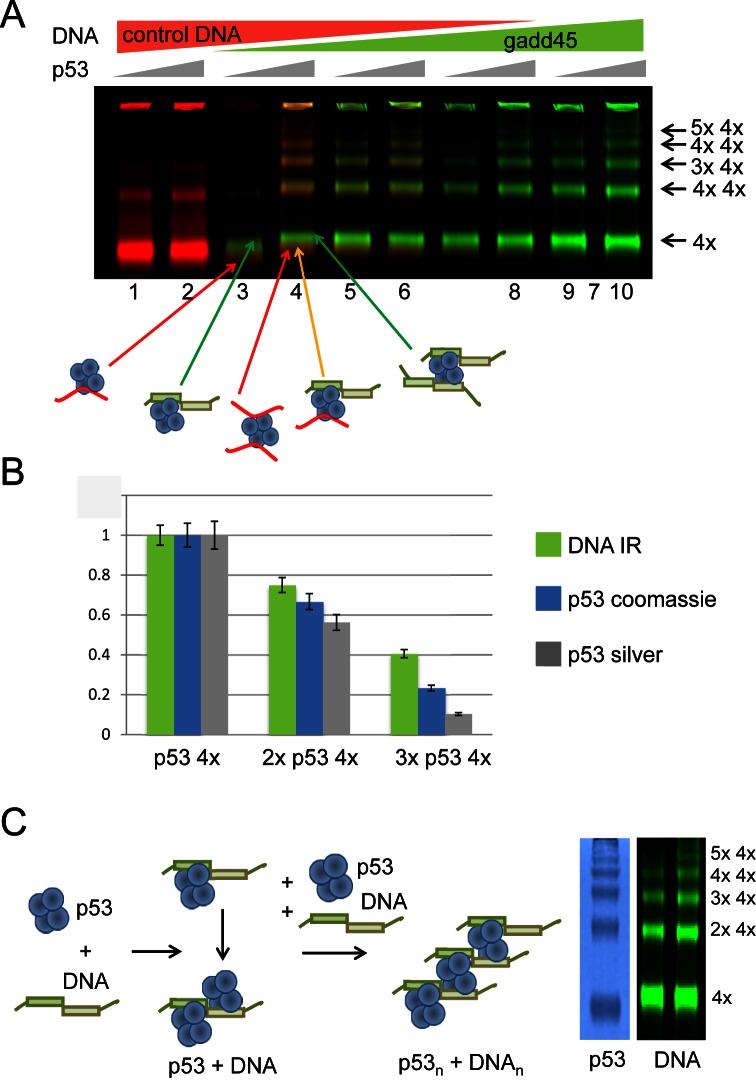
p53-DNA binding experiments with the IR dye-labeled DNA targets. (**A**) p53-DNA complexes were formed with two competing DNA targets – non-specific (control DNA) and specific, gadd45 RE DNA. Each strand of the non-specific DNA was labelled with IR700 dye (red) and similarly gadd45 DNA was labelled with IR800 dye (green). An increasing amount of specific DNA target was added to p53-DNA mixtures. Increasing amounts of p53 were used for each pair of lanes. All complexes were cross-linked with 0.025% GA and separated on 4–12% MOPS SDS PAGE. 100% of non-specific target (lanes 1 and 2) promoted only a small amount of double p53 tetramer binding to DNA, whereas the presence of specific DNA, even in small amounts, promoted efficient DNA-binding of multiple p53 tetramers (lanes 3–6), which was highest when only the specific DNA target was used (lanes 9 and 10). (**B**) Relative distribution of p53/DNA tetramers, double tetramers and triple tetramers. Density signals for different p53-DNA complexes were calculated by density scanning of IR-signal for IR-dye-labeled DNA and image density analysis for coomassie- or silver-stained p53 gels using the same set of p53-DNA binding reaction samples. Results are presented as ratios of normalized signal intensity. The typical distribution observed for p53 tetramers in complex with DNA was such that compared to the single p53 tetramer-DNA complexes there was ∼2/3 of that of the double p53 tetramer-DNA complexes and ∼1/3 of that of the triple p53 tetramer-DNA complexes. (**C**) A schematic model of p53-DNA complexes formed by p53 tetramers binding to the DNA RE targets. Two half-sites of p53 DNA RE are required to form high molecular order complexes between p53 and DNA. Multiples of p53 tetramer form an array interlaid with DNA.

To understand whether all p53 tetramers in the high order complexes are bound to the DNA targets we used DNA targets labeled with infrared (IR) dyes: IR700 dye-labeled nonspecific dsDNA (in red) and IR800 dye-labeled specific dsDNA (gadd45, in green) (Table 1; Figure 4A).

A combination of IR dye-labeled DNA targets in p53-DNA binding experiments and glutaraldehyde-fixed p53-DNA complex resolving on protein SDS-PAGE proved to be more efficient and convenient than the traditional DNA gel EMSA in determination of the molecular mass of the p53-DNA complexes. The p53-DNA complexes cross-linked with 0.025% glutaraldehyde were well resolved on the SDS PAGE and even a slight change in molecular mass due to addition of an extra dsDNA target (∼12 kDA) was clearly seen (Figure 4A). Furthermore, p53 appears to slightly change its conformation when bound to specific DNA, which was indicated by the difference in migration for p53 complexed with gadd45 and control non-specific DNA targets (Figure 4A, p53 tetramer-DNA complexes in green and red, respectively). The intensity of the IR800 dye in different p53-DNA complexes compared to data obtained by scanning coomassie and silver-staining of these gels showed that the amounts of p53 and DNA are proportional in all complexes (Figure 4B and C).

**Figure 5.**
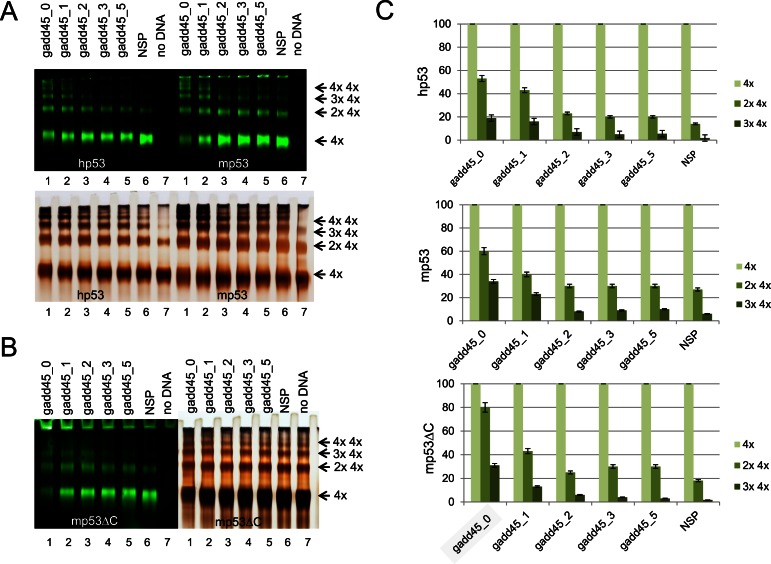
p53 multiple tetramers binding to DNA is most efficient when there is no spacer between half-sites. DNA targets containing different spacer length between two gadd45 RE half-sites were used to test their ability to promote high molecular order p53-DNA complexes. (**A**) Both human and murine p53 form multiple complexes on DNA targets with none (0 b) or one (1 bp) spacer between half-site sequences (gadd45_0 and gadd45_1, respectively) as visualized from gel shifts where p53 was bound to IR dye-labeled DNA targets followed by silver-staining to visualize proteins. (**B**) Similar results were obtained with murine mpΔ30 truncated protein lacking the last 30 amino acids of the C-terminal domain. (**C**) IR signal ratio between various p53 tetramer complexes (one, two and three tetramers) with different DNA targets was quantified to support the visual data in (A) and (B).

Using IR dye-labeled DNA allowed us to measure the difference in p53 tetramer binding on targets with different length of spacer between RE half-sites derived from gadd45 promoter (Supplementary Table S2). We used DNA target without spacer (gadd45_0) and four DNA targets with spacers of 1, 2, 3 and 5 bp. As a negative control we used non-specific DNA target similar to DNA20. Consistent with previous results (Figure 3 and Supplementary Figure S3) the best DNA targets to stimulate multiple p53 tetrameric complexes were those with two half-sites adjacent to each other (Figure 5A). Thus total lack of spacer (gadd45_0) or spacer of 1 bp length were the most efficient for both murine and human p53 multiple tetramer DNA binding.

Typically the amount of double p53 tetramers complexed with DNA was about 55–60% of the amount of single p53 tetramers when DNA target had no spacer between RE half-sites (gadd45_0). This proportion was decreased to ∼40% when spacer was 1 bp, followed by a sharp drop to ∼25% when spacer was 2 bp which was just above values obtained with DNA targets having spacer of 3 and 5 bp or the non-specific DNA target (Figure 5C). Similar data were obtained when the truncated versions of mp53 and hp53 lacking last 30 amino acid residues (mp53Δ30 and hp53Δ30) were tested and reactions contained either Tris or HEPES (Figure 5B and Supplementary Figure S4). Interestingly both murine and human Δ30 derivatives showed greater tendency to the p53Δ30 tetramer multimerization even without the presence of a DNA target as witnessed by silver staining of proteins (Figure 5B). However, analysis of data obtained by IR scans showed that these C-terminal truncates behaved similarly to the full-length p53, and only gadd45_0 and gadd45_1 DNA targets were efficiently bound by those multimeric complexes (Figure 5B and C). The efficiency of the double tetramer formation for p53Δ30 proteins was 10% and 20% higher for human and murine p53Δ30 proteins compared to the respective full-length p53 proteins.

To further examine a role of the tetrameric organization of p53 in multiple p53 tetramer/DNA complex formation we used murine p53 mutant with the double substitution M340Q/L344R. This mutant p53 protein was reported to form only dimers (55,56). Indeed, the dimeric p53 M340Q/L344R formed only a small amount of tetrameric complexes in solution with a dimeric assembly being a predominant form (Supplementary Figure S5). The fact that mutated p53 has failed to facilitate oligomerization on both specific and non-specific DNA targets implies that tetrameric p53 architecture is required for multiple binding to DNA RE (Supplementary Figure S5). This observation was further corroborated by experiments with the cancer-associated DNA-contact mutant of p53-R273H. Thus, the DNA-binding deficient mutant formed tetramers as expected but failed to form complexes on both specific and non-specific DNA targets (Supplementary Figure S6).

### Electron microscopy analysis of p53-DNA complexes

**Figure 6.**
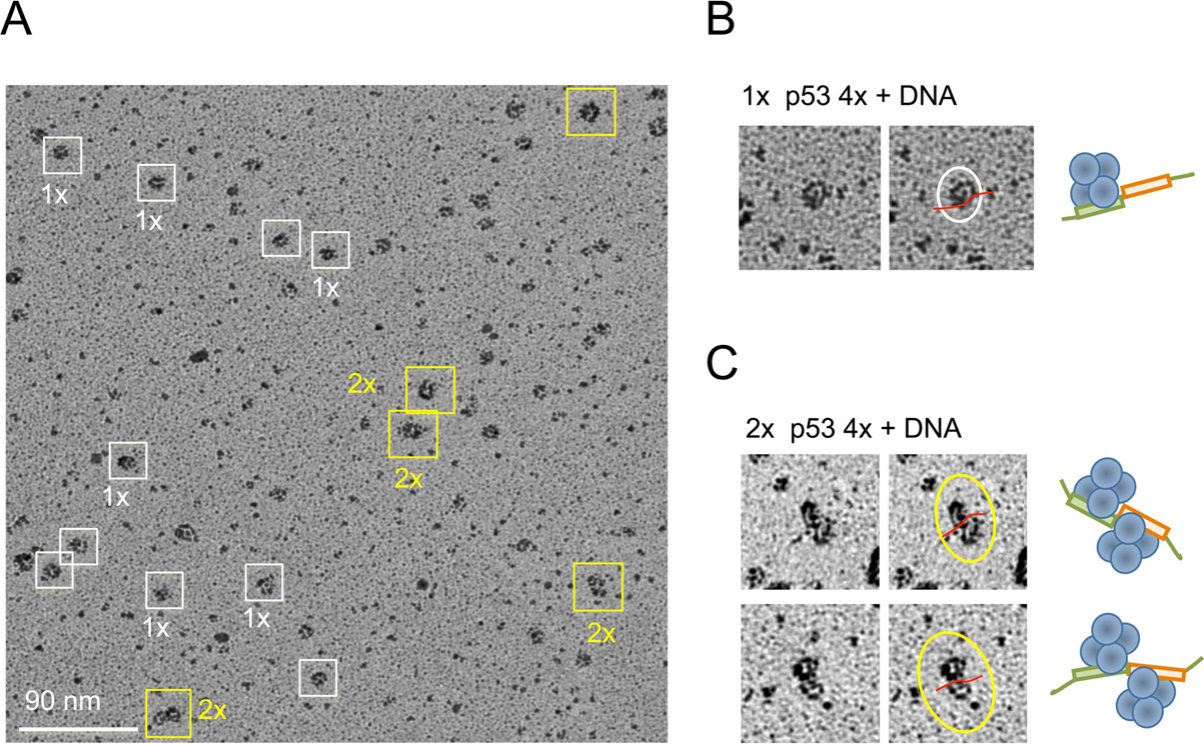
Electron microscopy of p53-DNA complexes. (**A**) Murine p53-DNA (gadd45, 20 bp) complexes cross-linked with 0.025% GA (sample shown on Figure 3D, lane 8) were analyzed by Pt-Ir rotary shadowing. White and yellow boxes highlight p53 tetramer–DNA and p53 double tetramer–DNA complexes, respectively. Selected images are shown on right side. DNA (red line) is seen in complex with single p53 tetramers (top right) (**B**) and two p53 tetramers (bottom right), where two tetramers are seen to bind to one DNA molecule, on opposite sides (**C**).

The gel-shift analysis demonstrated that the DNA targets with two immediately adjacent half-sites were the most efficient in promoting the multiple binding of p53 tetramers. We thus used complexes formed on the gadd45 DNA target (20 bp) to be visualized by electron microscopy using rotary shadowing (Figure 6). Images of p53-DNA complexes showed DNA-bound p53 particles that represented one p53 tetramer bound to DNA (Figure 6A, boxed in white) and two p53 tetramers bound to one DNA RE (Figure 6A, boxed in yellow). DNA RE could be clearly seen in complex with one p53 tetramer (Figure 6B) and two p53 tetramers (Figure 6C).

We next showed that the cross-linked p53-DNA complexes could also be well resolved on the native protein PAGE (Supplementary Figure S7) prompting us to consider extraction of specific complexes from the native gels and using them for electron microscopy image analysis via a modified gel-to-grid transfer method (57). The p53-DNA complexes resolved by electrophoresis in native conditions were visualized by using the IR dye-labeled specific DNA target (gadd45, 20 bp), then the gel zones containing complexes of interest such as single, double and triple p53 tetramer–DNA complexes were excised from the gel and blotted onto EM grids (Supplementary Figure S8 and Materials and Methods). The images of single, double and triple p53 tetramer–DNA complexes were selected from the micrographs of negatively stained samples (Supplementary Figure S8B).

**Figure 7.**
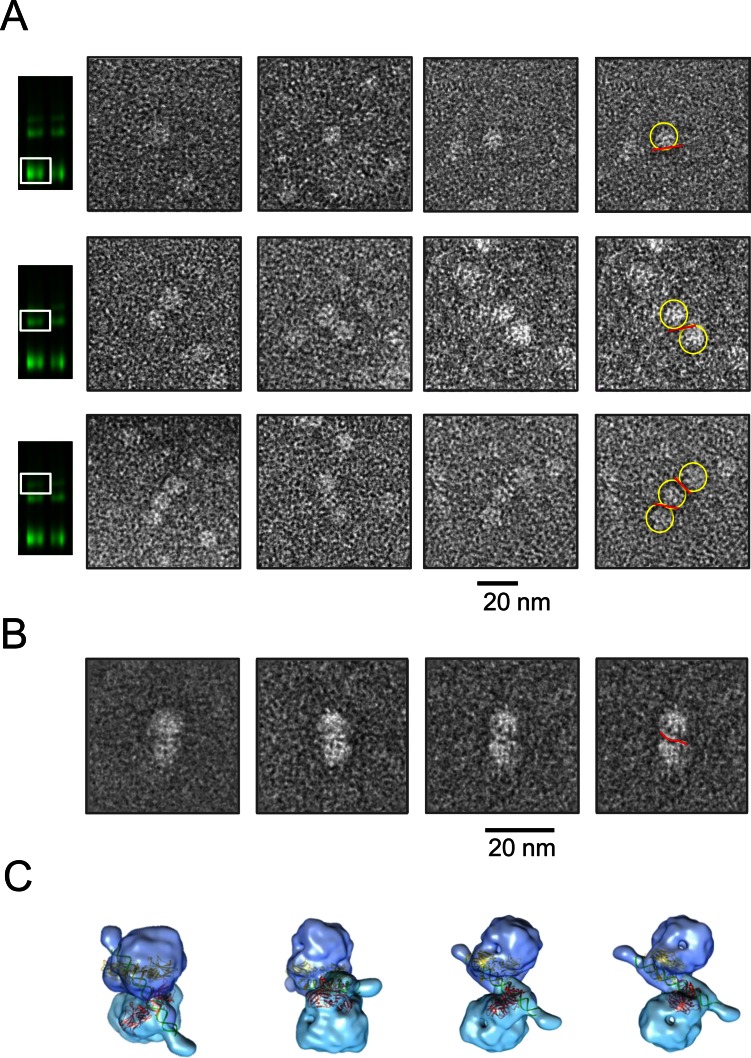
Image analysis of p53-DNA complexes. (**A**) Images of p53-DNA complexes obtained by the gel-to-grid method and EM analysis. Top row – complexes containing one p53 tetramer bound to DNA. The second row shows particles of p53 double tetramer–DNA complexes. The third row shows particles of p53 triple tetramer–DNA complexes. p53 tetramers are highlighted with yellow circles and DNA with red lines. (**B**) Representative class averages of p53 double tetramer–DNA complexes show two p53 tetramers bound to one DNA RE (gadd45). DNA is indicated in red in the far right image. (**C**) Models of two p53 tetramers bound to one DNA RE representing respective views in (B). Two cores pairs bound to half-site sequences (1ata, (36)) are shown in red and yellow, and DNA is shown in green.

The majority of particles from the gel-section corresponding to one tetramer of p53 bound to DNA were indeed single p53 tetramers, some of which had visible DNA bound to them (Figure 7A, top row). Similarly, the majority of particles from the double tetramer–DNA complexes were represented by two p53 tetramers with DNA sandwiched in between of them (Figure 7A, second row). Triple tetramer–DNA complexes though enriched were less frequent in the corresponding gel sections and the grid had a mixture of double and triple tetramers bound to DNA due to insufficient distance between p53-DNA complexes on the native gel in this area (Figure 7A, third row).

The representative class averages of p53 double tetramer–DNA complexes obtained by the single particle analysis of ∼500 selected particles show two p53 tetramers bound to one DNA RE (Figure 7B). The models corresponding to the respective classes were obtained by combining two EM 3D maps of p53 tetramers in complex with DNA (EMD-1896 (32)) and the crystal structure of two core domains bound to the RE half-site (1ata (40)). The fitted core domains are shown in red and yellow, and the general path of DNA is shown in green (Figure 7C).

## DISCUSSION

Tumor suppressor p53 is a transcriptional activator that regulates expression of genes, products of which decide the outcome of the cellular response to stress. The gene transcription activation is achieved by p53 binding to its specific DNA RE sequences (37,58,59). Much has been studied about this process, however the elucidation of the precise mechanism of how p53 tetramers bind RE DNA was hindered by the lack of structural information about the full-length p53 complexed with DNA.

To elucidate the mode by which p53 tetramers bind DNA RE we cross-linked p53-DNA complexes with GA and resolved them on denaturing and native protein gels. Complexes analyzed by native gels were visualized by the IR-labeled DNA and transferred directly onto EM grids for further image analysis. We believe that this combination of methods, used to visualize specific p53-DNA complexes has a broad potential for use in future studies of DNA binding proteins in complex with various DNA targets. Using this approach we demonstrated that for both human and murine p53 one DNA RE element promotes binding of two p53 tetramers in a sequence-specific manner. The double p53 tetramer binding was only efficient when the p53-specific DNA target consisted of two or more decameric half-sites with 0 or 1 bp spacer in between them. No binding of multiple p53 tetramers was observed for either dimeric p53 or DNA-binding deficient p53 proteins.

**Figure 8.**
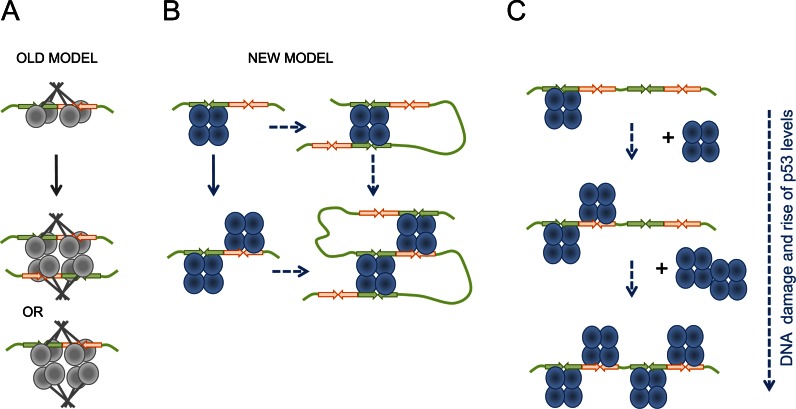
Biological implications of the multiple p53 tetramers DNA RE binding. Schematic representation of possible biological implications for double p53 tetramer binding to RE. (**A**) Old model of p53 tetramer interaction with DNA RE with a possible interaction between two p53 tetramers via protein interactions that is not mediated by DNA RE. (**B**) New model of p53 tetramer interaction with DNA RE based on our data. One tetramer bound to a half-site of the RE leaves the other half-site unoccupied providing an opportunity for a second p53 tetramer bind the remaining half-site and form the two p53 tetramers complex with one DNA RE. Either complex serves as a platform for DNA looping by utilizing free core domains and facilitating more efficient transcription activation. The ability to bind either side of the RE increases the probability of p53 finding it within the genomic context. (**C**) The consequent binding of p53 tetramers to DNA RE containing two or more half-sites can serve as a dose-dependent response in response to genotoxic stress and rising levels of p53, providing another level of p53-dependent transcriptional regulation.

Similar DNA-binding by multiples of p53 tetramers, specific to the full length p53, has been reported in earlier studies that used chemical cross-linking by GA and EM visualization (9,10,12,13). The observations were then interpreted as a stack of p53 tetramers perpendicular to DNA, within which only one p53 tetramer was bound to DNA, the model based on the crystallographic data of the isolated p53 core-DNA complex (Figure 8A) (12,13). However, no explanation was provided as to why the p53 tetramers could be bound together in a stack-like manner.

Our combined biochemical and EM image analysis of p53 tetramers bound to DNA RE showed that the mode of interaction is different to that previously suggested (22,40,43). Our results demonstrate that two p53 tetramers can interact sequence-specifically with one DNA RE. The biochemical and electron microscopy image analysis showed that the DNA target is positioned between two p53 tetramers, which occupy one half-site of RE each (Figure 7 and Supplementary Video). Thus, unlike the previously suggested mode of interaction the p53 tetramers are positioned on the opposite side of the DNA molecule and each tetramer is bound to one RE half-site essentially making the decameric half-site, a prime binding sequence for p53 tetramer (Figure 8B). This newly observed mode of interaction is consistent with our previous EM structural data that one p53 tetramer forms specific complex with DNA by occupying only one half-site of the DNA RE (32).

Our new data is also in agreement with previous reports that p53 can regulate transcription from non-canonical DNA REs (60,61). p53 REs comprised from 3/4 of the consensus RE (one half-site and one quarter site) and only those from one half-site were shown to be functional *in vivo* (37,60,61). The non-canonical p53 REs are less efficient and appear to require higher levels of p53 for transactivation of downstream genes. Their efficiency was shown to be on a par with p53 REs where two canonical half-sites are separated by spacers longer than 5 bp (61). Importantly, the most functionally efficient canonical p53 REs with two adjacent half-sites (spacer <2 bp) such as REs from *p21* and *gadd45* genes are also most efficient in double p53 tetramer binding as we have shown in this work. Moreover, the p53 RE from *TIGAR* gene was more efficient in stimulating multiple p53 tetramer DNA-binding when its natural 2 bp spacer between two half-sites was removed (Supplementary Figure S3).

This suggests a direct link between the structure of the p53 RE, its ability to promote double p53 tetramer DNA-binding and its transcriptional regulatory efficiency *in vivo*. It also indicates that the RE half-site is the prime working block for the tetrameric p53 RE DNA interaction and the number and sequence conservation of half-sites define efficiency and functionality of REs.

The results obtained raise the question why p53 RE has evolved to have two or more decameric half-sites. It is known that at least one half-site of RE is needed for an initial contact with p53 tetramer which makes it easier for p53 to locate its RE sites within the chromatin context. Once the first p53 tetramer is bound to RE it may help to recruit the second p53 tetramer to form two p53 tetramers per RE complex. It can also recruit the chromatin remodeling machinery, which p53 is known to interact with. When the RE is fully accessible it increases the probability p53 will bind either part of the RE in genome. Having two p53 tetramers bound to the RE would also provide a higher probability of DNA looping since either tetramer can now be involved in linking together distal REs (Figure 8B). In addition, having two p53 tetramers bound to one DNA RE would increase the chances of recruiting transcription co-factors needed for gene expression. We believe that the multiple p53 tetramer DNA-binding we describe here provides the answer to the question and highlights the biological implications of this p53 DNA-binding mode (Figure 8).

Interestingly the efficiency of multiple p53 tetramers binding to DNA appears to be at its best when two half-sites are immediately adjacent to each other. The ability of multiple p53 tetramers to bind to DNA was less efficient when the spacer separating half-sites was longer than 1 bp, but was more efficient again when the spacer was 15 bp-long. Moreover, inserting spacer longer than 1 bp into the *gadd45*-derived DNA target led to decrease of its ability to stimulate multiple p53 tetramers complex formation for both murine and human p53 proteins (Figure 5 and Supplementary Figure S4). This is consistent with a recent report on experiments *in vivo* that p53 REs are most efficient when their canonical half-sites are separated by less than 2 bp and show a dramatic drop in efficiency when their half-sites are separated by 5 bp or more (61). In this light it would be tempting to predict that some tetramer to tetramer contacts could stabilize the overall complex of two p53 tetramers on the two adjacent RE half-sites and that some post-translational modifications of p53 may fine-tune such interactions.

The basic C-terminal domain of p53 has been implicated in providing for p53's complexes with non-specific DNA and stabilizing p53 complexes with it specific REs (23–30). Here, we tested human and murine p53 constructs lacking the last C-terminal 30 amino acids (p53Δ30) known to be responsible for those functions. Interestingly, the removal of the basic C-terminal domain did not affect p53's ability to form multi-tetrameric complexes on DNA RE targets (Figure 5 and Supplementary Figures S2 and S4) indicating that the C-terminal regulatory domain of p53 does not provide for the p53 multiple tetramer DNA-binding.

In addition, the p53R273H cancer-derived mutant that does not to bind p53 DNA RE specifically but retains the non-specific DNA-binding failed to form multi p53 tetramer complexes with DNA. Thus, confirming that the non-specific DNA-binding of p53 does not contribute to the multiple binding of p53 tetramers to DNA REs (Supplementary Figure S6).

We have also demonstrated that the double p53 tetramer DNA-binding to RE depends on the intact tetrameric organization of p53. Thus, the dimeric M340Q/L344R p53 mutant was not capable of forming complexes of multiple p53 dimers on DNA targets despite its ability to bind p53 RE sequence specifically (55,56). Taken together with the fact that both human and murine p53 protein are tetramers in solution (Figure 1A and Supplementary Figure S1A) and our previous structural data (31,32), these new results confirm that the tetrameric assembly of p53 takes place before p53 binds its DNA RE and that tetramer is the functional unit of p53. This is also consistent with previous biophysical data demonstrating that p53 tetramer is the fundamental active unit of p53 (14,62).

The efficient binding of p53 tetramers to the DNA RE also appears to depend on the RE primary sequence. Thus, murine p53 tetramers formed double tetramer complexes less efficiently than their human counterparts when canonical human p21 and gadd45 REs were used, suggesting that p53 tetramers acquire subtle conformational changes once bound to DNA allowing them to stabilize the joint complex. The data are in agreement with a report that efficiency of p53 REs in transcriptional regulation is species-specific (63).

One tetramer per half-site of RE DNA-binding mode also supports the model of p53 level-dependent RE-binding and p53 target genes promoter regulation suggested and discussed previously (61,63–65). The binding of p53 tetramers to DNA RE containing two and more half-sites may serve for a rheostat-like p53 dose-dependent activation of specific genes in response to genotoxic stress and rising levels of p53, providing another level of p53-dependent transcriptional regulation (Figure 8C). Thus, promoters that have REs with two half-sites would need less (only two) p53 tetramers to fully activate them and promoters with REs that have more than two decameric half-sites such as *bax* and *mdm2* may need higher levels of p53 and more than two p53 tetramers to regulate their transactivation (63–65). This would be consistent with data that rising levels of p53 tetramers in the cell lead to higher rates of transactivation of the p53-regulated genes (66,67).

In addition, one could hypothesise that the joint binding of p53 tetramers to REs may allow for some degree of sequence degeneration due to potential cooperativity between p53 tetramers. Thus, degenerated half-sites within non-canonical REs could be a good target for p53 when the adjacent conserved half-sites have been already occupied by p53 tetramers. In such a case, those p53 REs will be fully functional at high levels of p53, e.g. the response from those REs will be more p53 dose-dependent than from REs that have two highly conservative half-site sequences with no spacer between them. These results suggest that there is an inherent adaptability in the recognition mechanism in which the less canonical p53 REs compensate for their sequence degeneration and large spacer length between half-sites by increasing numbers of half-sites in order to recruit more p53 tetramers.

Finally, due to the ability of p63 and p73 to bind p53 REs and transactivate p53 target genes and in agreement with reports that p63 and p73 contribute to a p53 response (68–73), some of the p53 family REs in our genome may serve as platforms for joint binding of the p53/p63/p73 family members to transcriptionally co-regulate downstream genes they share.

## Supplementary Material

SUPPLEMENTARY DATA

## References

[1] Vogelstein B., Lane D., Levine A.J. (2000). Surfing the p53 network. Nature.

[2] Oren M. (2003). Decision making by p53: life, death and cancer. Cell Death Differ..

[3] Murray-Zmijewski F., Slee E.A., Lu X. (2008). A complex barcode underlies the heterogeneous response to p53 to stress. Nat. Rev. Mol. Cell Biol..

[4] Vousden K.H., Prives C. (2009). Blinded by the Light: The Growing Complexity of p53. Cell.

[5] Vousden K.H., Lane D.P. (2007). p53 in health and disease. Nat. Rev. Mol. Cell Biol..

[6] Parant J.M., Lozano G. (2003). Disrupting TP53 in mouse models of human cancers. Hum. Mutat..

[7] Royds J.A., Iacopetta B. (2006). p53 and disease: when the guardian angel fails. Cell Death Differ..

[8] Brosh R., Rotter V. (2009). When mutants gain new powers: news from the mutant p53 field. Nat. Rev. Cancer.

[9] Stenger J.E., Mayr G.A., Tegtmeyer P. (1992). Formation of stable p53 homotetramers and multiples of tetramers. Mol. Carinog..

[10] Friedman P.N., Chen X., Bargonetti J., Prives C. (1993). The p53 protein is an unusually shaped tetramer that binds directly to DNA. Proc. Natl. Acad. Sci. U.S.A..

[11] Wang P., Reed M., Wang Y., Mayr G., Stenger J.E., Anderson M.E., Schwedes J.F., Tegtmeyer P. (1994). p53 domains: structure, oligomerisation and transformation. Mol. Cell. Biol..

[12] Stenger J.E., Tegtmeyer P., Mayr G.A., Reed M., Wang Y., Wang P., Hough P.V.C., Mastrangelo I.A. (1994). p53 oligomeristion and DNA looping are linked with transcriptional activation. EMBO J..

[13] Wang Y., Schwedes J.F., Parks D., Mann K., Tegtmeyer P. (1995). Interaction of p53 with its consensus DNA-binding site. Mol. Cell. Biol..

[14] Nicholls C.D., McLure K.G., Shields M.A., Lee P.W. (2002). Biogenesis of p53 involves cotranslational dimerization of monomers and post-translational dimerization of dimers. Implications on the dominant negative effect. J. Biol. Chem..

[15] Rajagopalan S., Huang F., Fersht A.R. (2011). Single-molecule characterization of oligomerization kinetics and equilibria of the tumor suppressor p53. Nucleic Acids Res..

[16] Joerger A.C., Fersht A.R. (2007). Structural biology of the tumor suppressor p53. Annu. Rev. Biochem..

[17] Okorokov A.L., Orlova E.V. (2009). Structural biology of the p53 tumour suppressor. Curr. Opin. Struct. Biol..

[18] Kern S.E, Kinzler K.W., Bruskin A., Jarosz D., Friedman P., Prives C., Vogelstein B. (1991). Identification of p53 as a sequence-specific DNA-binding protein. Science.

[19] el-Deiry W.S, Tokino T., Velculescu V.E., Levy D.B., Parsons R., Trent J.M, Lin D., Mercer W.E., Kinzler K.W., Vogelstein B. (1993). WAF1, a potential mediator of p53 tumor suppression. Cell.

[20] Wang B., Xiao Z., Ren E.C. (2009). Redefining the p53 response element. Proc. Natl. Acad. Sci. U.S.A..

[21] Harris C.R., Dewan A., Zupnick A., Normart R., Gabriel A., Prives C., Levine A.J., Hoh J. (2009). p53 responsive elements in human retrotransposons. Oncogene.

[22] Cho Y., Gorina S., Jeffrey P.D., Paveltich N.P. (1994). Crystal structure of p53 tumour suppressor-DNA complex: understanding tumorigenic mutations. Science.

[23] Lee S., Elenbaas B., Levine A., Griffith J. (1995). p53 and its 14 kDa C-terminal domain recognize primary DNA damage in the form of insertion/deletion mismatches. Cell.

[24] Bakalkin G., Selivanova G., Yakovleva T., Kiseleva E., Kashuba E., Magnusson K.P., Szekely L., Klein G., Terenius L., Winkman K.G. (1995). p53 binds single-stranded DNA ends through the C-terminal domain and internal DNA segments via the middle domain. Nucleic Acids Res..

[25] Ahn J., Prives C. (2001). The C-terminus of p53: the more you learn the less you know. Nat. Struct .Biol..

[26] McKinney K., Prives C. (2002). Efficient specific DNA binding by p53 requires both its central and C-terminal domains as revealed by studies with high-mobility group 1 protein. Mol. Cell. Biol..

[27] McKinney K., Mattia M., Gottifredi V., Prives C. (2004). p53 linear diffusion along DNA requires its C terminus. Mol. Cell.

[28] Hamard P.J., Lukin D.J., Manfredi J.J. (2012). p53 basic C terminus regulates p53 functions through DNA binding modulation of subset of target genes. J. Biol. Chem..

[29] Kim H., Kim K., Choi J., Heo K., Baek H.J., Roeder R.G., An W. (2012). p53 requires an intact C-terminal domain for DNA binding and transactivation. J. Mol. Biol..

[30] Laptenko O., Shiff I., Freed-Pastor W., Zupnick A., Mattia M., Freulich E., Shamir I., Kadouri N., Kahan T., Manfredi J. (2015). The p53 C terminus controls site-specific DNA binding and promotes structural changes within the central DNA binding domain. Mol. Cell.

[31] Okorokov A.L, Sherman M.B., Plisson C., Grinkevich V., Sigmundsson K., Selivanova G., Milner J., Orlova E.V. (2006). The structure of p53 tumour suppressor protein reveals the basis for its functional plasticity. EMBO J..

[32] Aramayo R., Sherman M.B., Brownless K., Lurz R., Okorokov A.L., Orlova E.V. (2011). Quaternary structure of the specific p53-DNA compelx reveals the mechanism of p53 mutant dominance. Nucleic Acids Res..

[33] Tokino T., Thiagalingam S., el-Deiry W.S., Waldman T., Kinzler K.W., Vogelstein B. (1994). p53 tagged sites from human genomic DNA. Hum. Mol. Genet..

[34] Kim E., Deppert W. (2006). The versatile interactions of p53 with DNA: when flexibility serves specificity. Cell Death Differ..

[35] Wei C.L., Wu Q., Vega V.B., Chiu K.P., Ng P., Zhang T., Shahab A., Yong H.C., Fu Y, Weng Z. (2006). A global map of p53 transcription-factor binding sites in the human genome. Cell.

[36] Smeenk L., van Heeringen S.J., Koeppel M., van Driel M.A., Bartels S.J., Akkers R.C., Denissov S., Stunnenberg H.G., Lohrum M. (2008). Characterization of genome-wide p53-binding sites upon stress response. Nucleic Acids Res..

[37] Menendez D., Inga A., Resnick M.A. (2009). The expanding universe of p53 targets. Nat. Rev. Cancer.

[38] McLure K.G., Lee P.W. (1998). How p53 binds DNA as a tetramer. EMBO J..

[39] Zhao K., Chai X., Johnston K., Clements A., Marmorstein R. (2001). Crystal structure of the mouse p53 core DNA-binding domain at 2.7 A resolution. J. Biol. Chem..

[40] Kitayner M., Rozenberg H., Kessler N., Rabinovich D., Shaulov L., Haran T.E., Shakked Z. (2006). Structural basis of DNA recognition by p53 tetramers. Mol. Cell.

[41] Chen Y., Dey R., Chen L. (2010). Crystal structure of the p53 core domain bound to a full consensus site as a self-assembled tetramer. Structure.

[42] Kitayner M., Rozenberg H., Rohs R., Suad O., Rabinovich D., Honig B., Shakked Z. (2010). Diversity in DNA recognition by p53 revealed by crystal structures with Hoogsteen base pairs. Nat. Struct. Mol. Biol..

[43] Shakked Z. (2007). Quaternary structure of p53: the light at the end of the tunnel. Proc. Natl. Acad. Sci. U.S.A..

[44] Melero R., Rajagopalan S., Lázaro M., Joerger A.C., Brandt T., Veprintsev D.B., Lasso G., Gil D., Scheres S.H., Carazo J.M. (2011). Electron microscopy studies on the quaternary structure of p53 reveal different binding modes for p53 tetramers in complex with DNA. Proc. Natl. Acad. Sci. U.S.A..

[45] Hyatt M.A. (2000). Principles and techniques of electron microscopy: biological applications.

[46] Ludtke S.J., Baldwin P.R., Chiu W. (1999). EMAN: semiautomated software for high-resolution single-particle reconstructions. J. Struct. Biol..

[47] Mindell J.A., Grigorieff N. (2003). Accurate determination of local defocus and specimen tilt in electron microscopy. J. Struct. Biol..

[48] van Heel M., Harauz G., Orlova E.V., Schmidt R., Schatz M. (1996). A new generation of the IMAGIC image processing system. J. Struct. Biol..

[49] Pettersen E.F., Goddard T.D., Huang C.C., Couch G.S., Greenblatt D.M., Meng E.C., Ferrin T.E. (2004). UCSF Chimera - a visualization system for exploratory research and analysis. J. Comput. Chem..

[50] Kastan M.B., Zhan Q., el-Deiry W.S., Carrier F., Jacks T., Walsh W.V., Plunkett B.S., Vogelstein B., Fornace A.J. (1992). A mammalian cell cycle checkpoint pathway utilizing p53 and GADD45 is defective in ataxia-telangiectasia. Cell.

[51] Zambetti G.P., Bargonetti J., Walker K., Prives C., Levine A.J. (1992). Wild-type p53 mediates positive regulation of gene expression through a specific DNA sequence element. Genes Dev..

[52] Wu X., Bayle J.H., Olson D., Levine A.J. (1993). The p53-mdm-2 autoregulatory feedback loop. Genes Dev..

[53] Miyashita T., Reed J.C. (1995). Tumor suppressor p53 is a direct transcriptional activator of the human bax gene. Cell.

[54] Bensaad K., Tsuruta A., Selak M.A., Vidal M.N., Nakano K., Bartrons R., Gottlieb E., Vousden K.H. (2006). TIGAR, a p53-inducible regulator of glycolysis and apoptosis. Cell.

[55] Lee W., Harvey T.S., Yin Y., Yau P., Litchfield D., Arrowsmith C.H. (1994). Solution structure of the tetrameric minimum transforming domain of p53. Nat. Struct. Biol..

[56] Davison T.S., Nie X., Ma W., Lin Y., Kay C., Benchimol S., Arrowsmith C.H. (2001). Structure and functionality of a designed p53 dimer. J. Mol. Biol..

[57] Knispel R.W., Kofler C., Boicu M., Baumeister W., Nickell S. (2012). Blotting protein complexes from native gels to electron microscopy grids. Nat. Methods.

[58] Riley T., Sontag E., Chen P., Levine A. (2008). Transcriptional control of human p53-regulated genes. Nat. Rev. Mol. Cell. Biol..

[59] Espinosa J.M. (2008). Mechanisms of regulatory diversity within the p53 transcriptional network. Oncogene.

[60] Menendez D., Krysiak O., Inga A., Krysiak B., Resnick M.A., Schönfelder G. (2006). A SNP in the flt-1 promoter integrates the VEGF system into the p53 transcriptional network. Proc. Natl. Acad. Sci U.S.A..

[61] Jordan J.J., Menendez D., Inga A., Noureddine M., Bell D.A., Resnick M.A. (2008). Noncanonical DNA motifs as transactivation targets by wild type and mutant p53. PLoS Genet..

[62] Weinberg R.L., Veprintsev D.B., Fersht A.R. (2004). Cooperative binding of tetrameric p53 to DNA. J. Mol. Biol..

[63] Jegga A.G., Inga A., Menendez D., Aronow B.J., Resnick M.A. (2008). Functional evolution of the p53 regulatory network through its target response elements. Proc. Natl. Acad. Sci. U.S.A..

[64] Jordan J.J., Menendez D., Sharav J., Beno I., Rosenthal K., Resnick M.A., Haran T.E. (2012). Low-level p53 expression changes transactivation rules and reveals superactivating sequences. Proc. Natl. Acad. Sci. U.S.A..

[65] Menendez D., Resnick M.A., Haran T. (2012). Transactivation by low and high levels of human p53 reveals new physical rules of engagement and novel super-transactivation sequences. Cell Cycle.

[66] Gaglia G., Lahav G. (2014). Constant rate of p53 tetramerization in response to DNA damage controls the p53 response. Mol. Syst. Biol..

[67] Gaglia G., Guan Y., Shah J.V., Lahav G. (2013). Activation and control of p53 tetramerization in individual living cells. Proc. Natl. Acad. Sci. U.S.A..

[68] Di Como C.J., Gaiddon C., Prives C. (1999). p73 function is inhibited by tumor-derived p53 mutants in mammalian cells. Mol. Cell. Biol..

[69] Gaiddon C., Lokshin M., Ahn J., Zhang T., Prives C. (2001). A subset of tumor-derived mutant forms of p53 down-regulate p63 and p73 through a direct interactionwith the p53 core domain. Mol. Cell. Biol..

[70] Flores E.R., Tsai K.Y., Crowley D., Sengupta S., Yang A., McKeon F., Jacks T. (2002). p63 and p73 are required for p53-dependent apoptosis in response to DNA damage. Nature.

[71] Lin Y.L., Sengupta S., Gurdziel K., Bell G.W., Jacks T., Flores E.R. (2009). p63 and p73 transcriptionally regulate genes involved in DNA repair. PLoS Genet..

[72] Ciribilli Y., Monti P., Bisio A., Nguyen H.T., Ethayathulla A.S., Ramos A., Foggetti G., Menichini P., Menendez D., Resnick M.A. (2013). Transactivation specificity is conserved among p53 family proteins and depends on a response element sequence code. Nucleic Acids Res..

[73] Kenzelmann Broz D., Spano Mello S., Bieging K.T., Jiang D., Dusek R.L., Brady C.A., Sidow A., Attardi L.D. (2013). Global genomic profiling reveals an extensive p53-regulated autophagy program contributing to key p53 responses. Genes Dev..

