# Supplementary material for: Two p53 tetramers bind one consensus DNA response element
Source: Nucleic Acids Res. 2016 Mar 31;44(13):6185–99. doi: 10.1093/nar/gkw215 (PMC5291249; doi:10.1093/nar/gkw215)
Supplement: SUPPLEMENTARY DATA [file supp_44_13_6185__index.html]

Two p53 tetramers bind one consensus DNA response element — Two p53 tetramers bind one consensus DNA response element — Two p53 tetramers bind one consensus DNA response element — SUPPLEMENTARY DATA 

# Two p53 tetramers bind one consensus DNA response element

## SUPPLEMENTARY DATA

- SUPPLEMENTARY DATA
- SUPPLEMENTARY DATA
