# Supplementary material for: Two p53 tetramers bind one consensus DNA response element
Source: Nucleic Acids Res. 2016 Mar 31;44(13):6185–99. doi: 10.1093/nar/gkw215 (PMC5291249; doi:10.1093/nar/gkw215)
Supplement: SUPPLEMENTARY DATA [file supp_gkw215_nar-00671-h-2016-File010.pdf]

SUPPLEMENTARY DATA

Supplementary Figures legends

Supplementary Figure 1. Murine and human p53 tetramers behave identically in forming high molecular order complexes on DNA RE. (**A**) Aliquots of the recombinant human p53 (left panel) and murine p53 (right panel) were crosslinked with increasing concentrations of glutaraldehyde (GA). Lane 1 - 0.01%; lane 2 – 0.025%; lane 3 – 0.05%; lane 4 – 0.1% and lane 5 – 0.5%) and products of reactions were separated on 4-12% MOPS SDS PAGE. (**B** and **C**) Complexes of the recombinant human p53 (left panel) and murine p53 (right panel) with dsDNA (DNA#1, 20bp) were cross-linked with GA (0.025%) and analysed by 4-12% MOPS SDS PAGE. Increasing amounts of p53 (0.25, 0.5, 1.25, 2.5 and 5 µg) were used in (B) and increasing amounts of DNA targets with 2 µg of p53 were used in (C). Products of crosslinking resolved in a ladder-like pattern of high molecular order complexes, molecular masses of which corresponded to one, two, three, four and higher amount of p53 tetramer per complex.

Supplementary Figure 2. Multiple binding of p53 tetramers to DNA RE in different reaction buffers and on longer DNA targets. p53 forms high molecular order complexes with specific DNA equally efficient in Tris and HEPES-based buffers and on longer DNA-targets. (**A**) 66 bp long DNA targets composed of different amount half-sites and quarter sites (Supplementary Figure 1). Quarter site sequence RRRCW depicted as → and quarter site sequence WGYYY depicted as ←, where R= purine, Y= pyrimidine, and W is either A or T. The complete half-site is →←. Different colours are used to show two adjacent half-sites. **~** - non-specific DNA sequence, which in case of DNA 21 represents flanking sequences of 23 bp on each side. (**B**) Recombinant p53 (murine) can only efficiently form high order molecular complexes with DNA targets that have both half-sites (DNA #21). p53 forms double tetramers in solution but it is not stimulated any further by less specific DNA targets #20, 22-25. The truncated version of mp53 lacking last 30 amino acid residues (mp53Δ30) showed results similar to those of the full-length

mp53 protein (lanes 9-15 compared top lanes 2-9, Suppl. Fig. 2B). Controls – two lanes 8 and 15, no DNA.

Supplementary Figure 3 DNA-binding of multiple p53 tetramers to REs with different spacer length. (**A**) DNA targets with different spacer length between the RE half-sites were used to test their ability to promote high molecular order p53-DNA complexes (Supplementary Figure 1). The half-sites depicted as →←. **~** - non-specific DNA sequence. (**B** and **C**) Both human and murine p53 form multiple complexes on DNA targets with different spacer length between the half-sites (DNA targets # 31-37). DNA targets without any or only 1bp spacer (DNA targets #21, 31 & Tigar_sp0) appear to be more efficient in stimulating high order complexes.

Supplementary Figure 4 p53 multiple tetramers binding to DNA is most efficient when there is no spacer between half-sites in different reaction buffers. DNA targets containing different spacer length between two gadd45 RE half-sites were used to test their ability to promote high molecular order p53-DNA complexes in reactions containing Tris or HEPES. Both full length human p53 (**A**) and its hpΔ30 truncated version lacking the last 30 amino acids of the C-terminal domain (**C**) formed multiple tetramers complexes efficiently on DNA targets with none (0b) or one (1bp) spacer between the half-site sequences (gadd45_0 and gadd45_1 respectively) as visualised from gel shifts with IR dye-labelled DNA targets. (**C** and **D**) IR signal ratio between various p53 tetramer complexes (one, two and three tetramers of hp53 or hpΔ30) with diferent DNA targets was quantified to support visual data in (A) and (B).

Supplementary Figure 5. Murine p53 double mutant M340Q/L344R protein that forms dimers (49, 50) was tested for its ability to form multiples of p53 dimers (or tetramers) on specific and non-specific DNA. (**A**) The dimeric p53 formed only a small amount of tetrameric complexes in solution (lane 12). The addition of specific and non-specific DNA targets (as in Figures 2 and 3) failed to promote this any further, leading to primarily

p53 dimer DNA complex thus indicating that intact tetrameric p53 architecture is required for multiple p53 tetramers binding to DNA RE. Lane 13 – positive control with mp53 complexed with DNA1. (**B**) Dimeric p53 was tested to form complexes with competing non-specific and specific (gadd45) DNA targets labelled with IR700 (red) and IR800 (green) dyes respectively. There were no large molecular complexes of high order formed as observed previously with tetrameric p53. Left and right panels show the same samples stained with either silver (for protein) or scanned for IR dye signal (for DNA) respectively. (**C**) Normalised ratios of p53/DNA dimers and double dimers. Density signals for different p53-DNA complexes were calculated by density scanning of IR-signal for IR-dye-labelled DNA. Results are presented as ratios of normalised signal intensity.

Supplementary Figure 6. Murine p53 cancer-associated "hotspot" mutant R273H was tested for its ability to form multiples of p53 tetramers on specific and non-specific DNA. p53 R273H was tested to form complexes with competing non-specific and specific (gadd45) DNA targets labelled with IR700 (red) and IR800 (green) dyes respectively. (**A**) There were no large molecular complexes of high order formed as it was observed previously with wild type p53. Left and right panels show the same samples stained with either silver (for protein) or scanned for IR dye signal (for DNA) respectively. p53 R273H formed tetramers in the absence of DNA targets and only very small amount of double tetramers in the presence of either specific or non-specific DNA targets indicating that ability to bind DNA sequence specifically is required for multiple p53 tetramers to bind p53 DNA RE. (**B**) Normalised ratios of p53 R273H /DNA tetramers and double tetramers. Density signals for different p53-DNA complexes were calculated by density scanning of IR-signal for IR-dye-labelled DNA. Results are presented as ratios of normalised signal intensity.

Supplementary Figure 7. Samples of p53-DNA complexes formed as described in Figure 5 using a competition between non-specific (IR700 dye-labelled, red) and specific (IR800 dye-labelled, green) DNA targets were cross-linked with 0.025% GA and resolved on either 4-12% MOPS SDS PAGE (top panel) or on native (blue) protein gel (bottom

panel). Non-specific DNA target did not promote the formation of high molecular complexes (in red) whereas specific DNA target (gadd45) did so (in green). Whenever the population of p53-DNA complexes contained both DNA targets the colours of two dyes combined to give orange. Complexes of p53 tetramers on DNA target were resolved sufficiently well on the native gel to use zone of the gel for transfer onto EM grids.

Supplementary Figure 8. p53-DNA complex transfer from native gel onto EM grid. The p53-DNA complexes resolved by electrophoresis in native conditions were visualised by using the IR dye- labelled specific DNA target (gadd45, 20bp), then the gel zones containing complexes of interest such as single, double and triple p53 tetramer-DNA complexes were excised from the gel and blotted onto EM grids. (**A**) p53-DNA complexes were separated on native 4-16% bis-tris gel and visualised using IR dye-labelled DNA target. The gel was scanned without removing the plastic cast and/or leaving gel on one of the plates to allow marking. Gel zones containing p53-DNA complexes of interest were selected and marked on the cast plate. The cast was opened and appropriate zones were cut off using a scalpel (white boxes). Each chosen section of the gel were placed on the EM copper grid (carbon film towards the gel) and another glass plate was placed on the top of the gel (schematically shown on the right side of the panel). To transfer the protein-DNA complex particles from the gel to the carbon grid with a weight press to allow protein-DNA complex particles diffusion onto the grid. Then the samples were negatively stained with 2% uranyl acetate. EM grids containing extracts from gel bands with p53-DNA complexes of interest were examined by EM followed by image analysis (**B**).

Supplementary Video.

Models of two p53 tetramers (EMDB-1896 map, ref. 28) bound to one DNA RE representing respective views in (Fig.7B). Two cores pairs bound to half-site sequences (1ata, ref. 36) are shown in red and yellow, and DNA is shown in green.

Supplementary Table 1. Oligonucleotide DNA targets used in p53 DNA-binding experiments for Supplementary Figures 2 and 3. Corresponding forward (fwd) and the complementary reverse (rev) oligonucleotides were annealed to each other produce the double- stranded DNA targets. DNA targets 21-25 were derived from DNA targets 1-5 (Table 1) with addition of extra flanking sequences to increase the overall length of the DNA targets to 66 bp. DNA 20 is fully nonspecific DNA target. All DNA sequences are shown as 5' → 3', spacer and flanking sequences within and up- and down-stream of p53 RE regions are in black, conserved specific sequences within p53 RE regions are in blue, nonspecific sequences within p53 RE regions are in red.

Supplementary Table 2. Oligonucleotide DNA targets used in p53 DNA-binding experiments for Figure 5 and Supplementary Figure 5. Corresponding forward (fwd) and the complementary reverse (rev) oligonucleotides were annealed to each other produce the double-stranded DNA targets. The overall length of the DNA targets was 34 bp. DNA 20 is fully nonspecific DNA target. All DNA sequences are shown as 5' to 3', spacer and flanking sequences within and up- and down-stream of p53 RE regions are in black, conserved specific sequences within p53 RE regions are in blue, nonspecific sequences within p53 RE regions are in red. All DNA targets were IR800-labeled at 5’ end, synthesized at 100nM scale and HPLC purified.

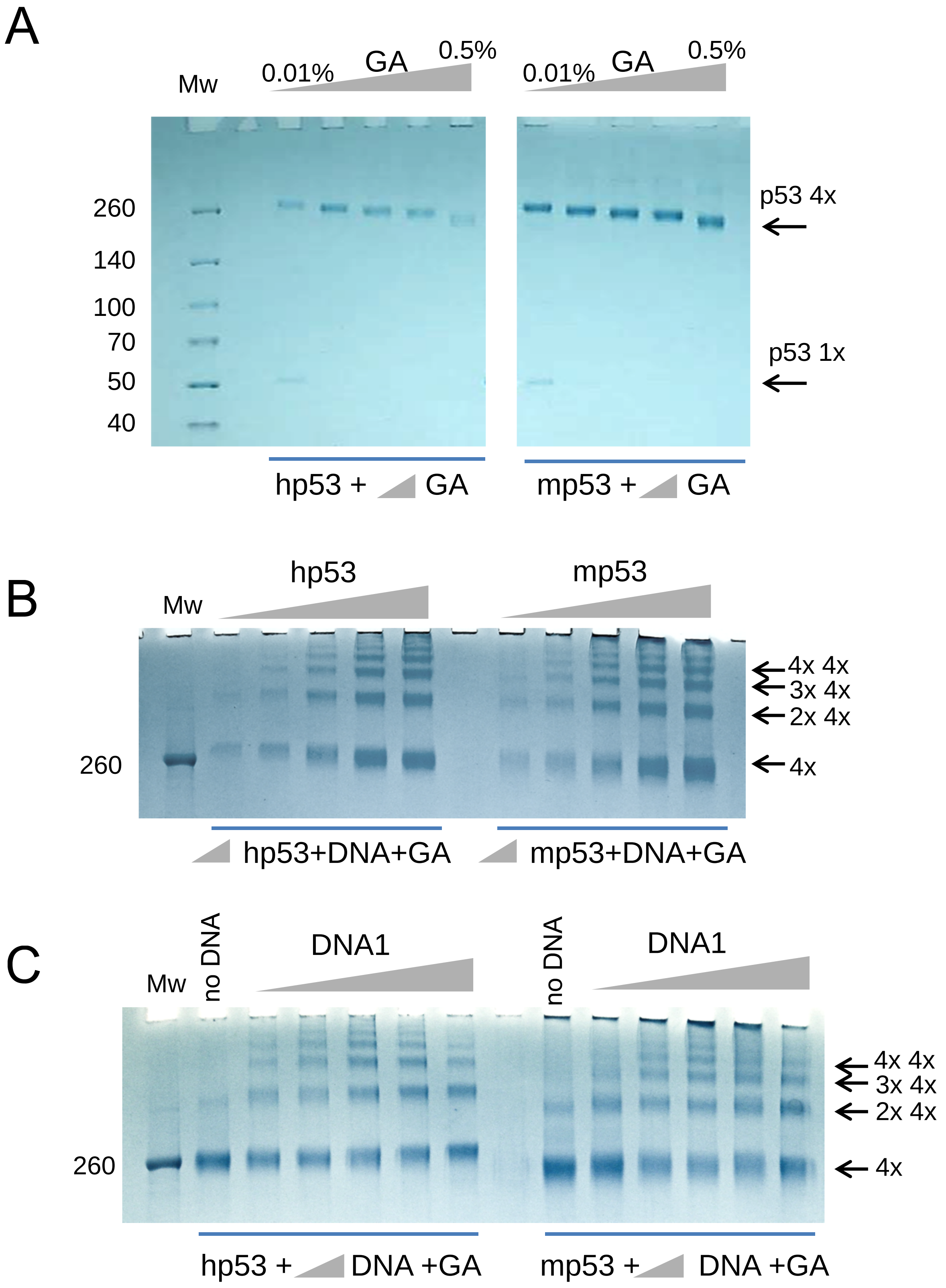
A
Mw
0.01%
GA
0.5%
0.01%
GA
0.5%
260
140
100
70
50
40
p53 4x
p53 1x
hp53 + GA
mp53 + GA
B
Mw
hp53
mp53
260
4x 4x
3x 4x
2x 4x
4x
hp53+DNA+GA
mp53+DNA+GA
C
Mw
no DNA
DNA1
no DNA
DNA1
260
4x 4x
3x 4x
2x 4x
4x
hp53 + DNA +GA
mp53 + DNA +GA

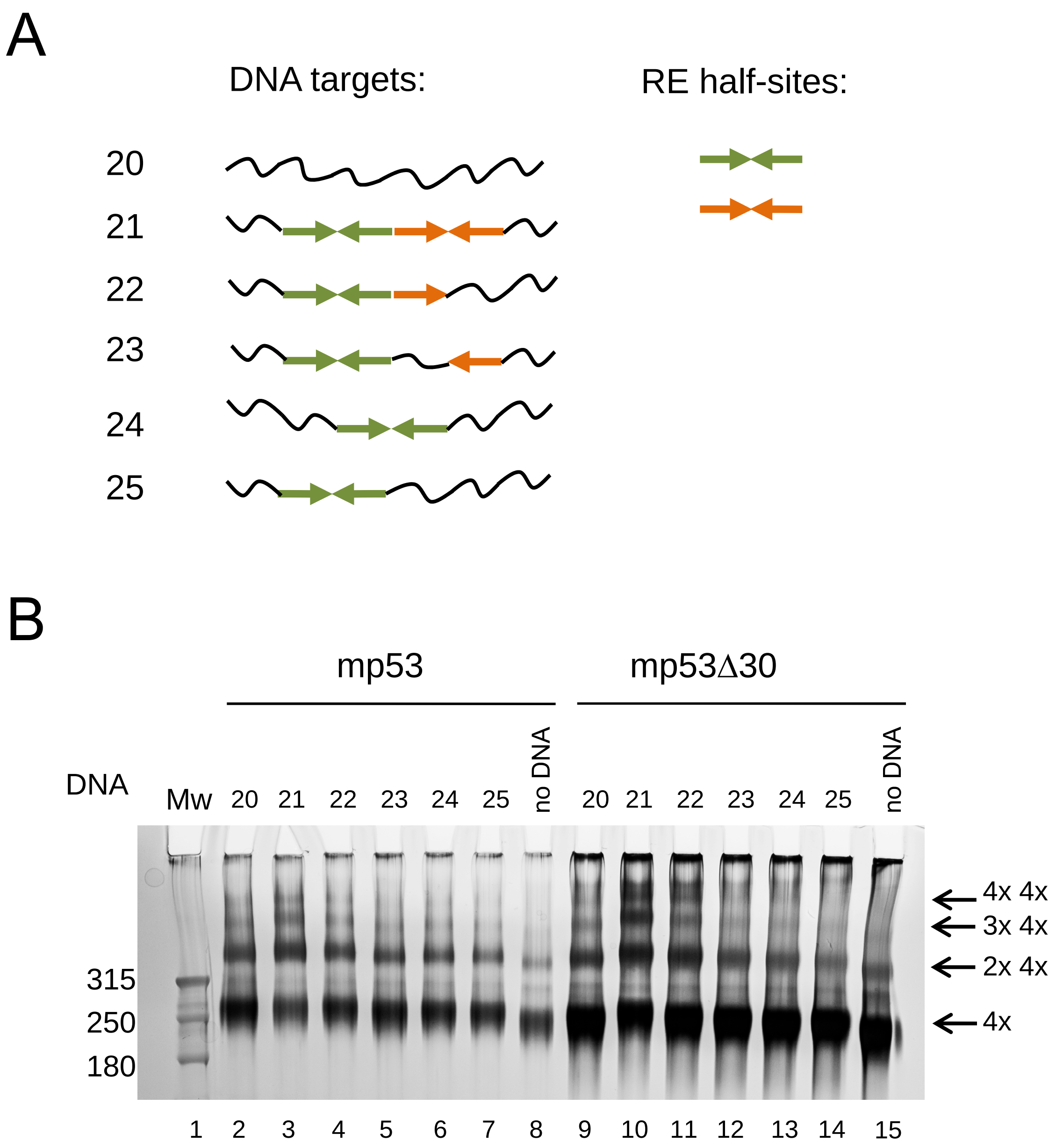
A
DNA targets:
RE half-sites:
20
21
22
23
24
25
B
mp53
mp53Δ30
DNA
Mw
20 21 22 23 24 25 no DNA
20 21 22 23 24 25 no DNA
315
250
180
4x 4x
3x 4x
2x 4x
4x
1 2 3 4 5 6 7 8 9 10 11 12 13 14 15

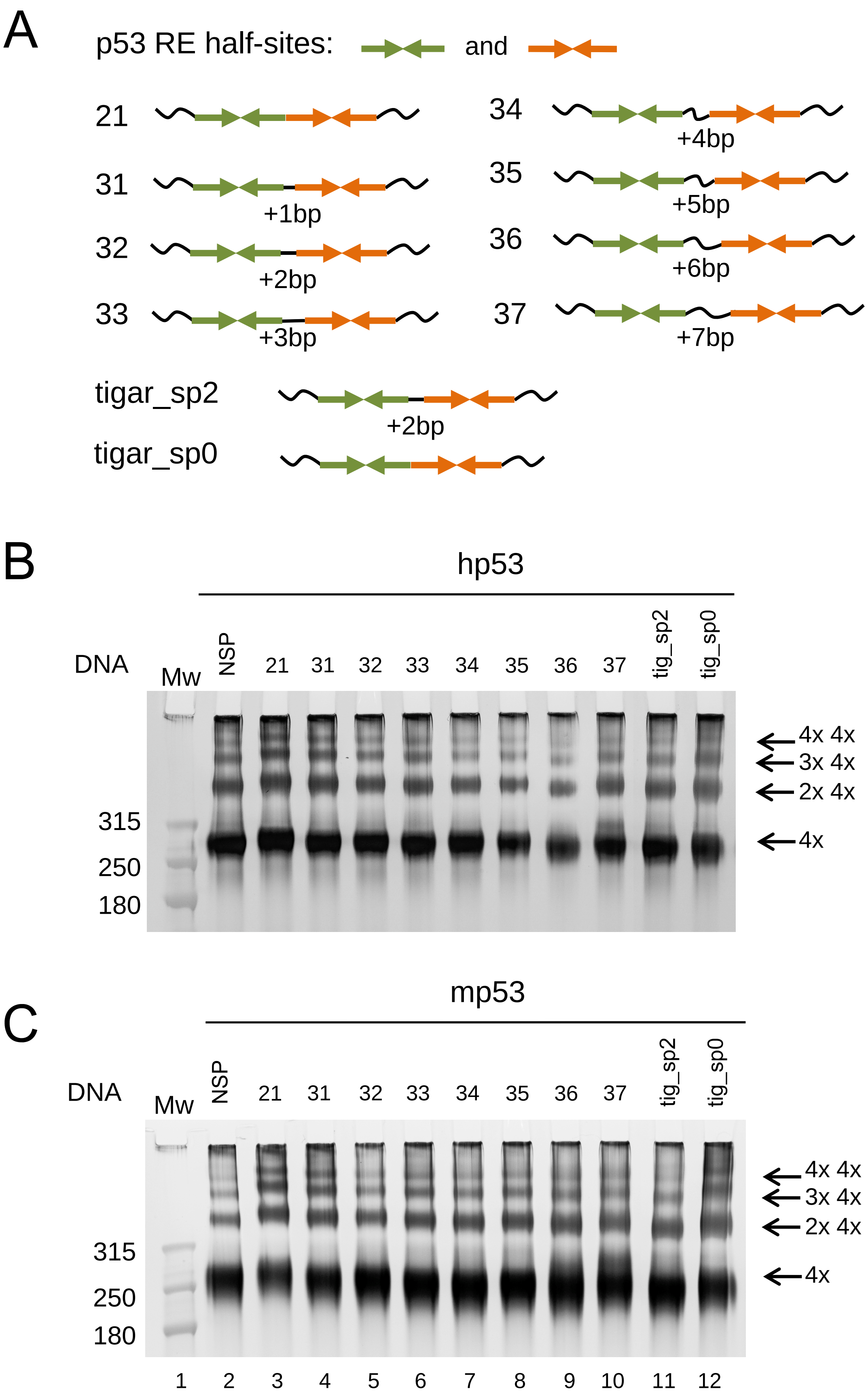
A
p53 RE half-sites:
and
21
31
+1bp
32
+2bp
33
+3bp
34
+4bp
35
+5bp
36
+6bp
37
+7bp
tigar_sp2
+2bp
tigar_sp0
B
hp53
DNA
Mw
NSP
21
31
32
33
34
35
36
37
tig_sp2
tig_sp0
315
250
180
4x 4x
3x 4x
2x 4x
4x
C
mp53
DNA
Mw
NSP
21
31
32
33
34
35
36
37
tig_sp2
tig_sp0
315
250
180
4x 4x
3x 4x
2x 4x
4x
1 2 3 4 5 6 7 8 9 10 11 12

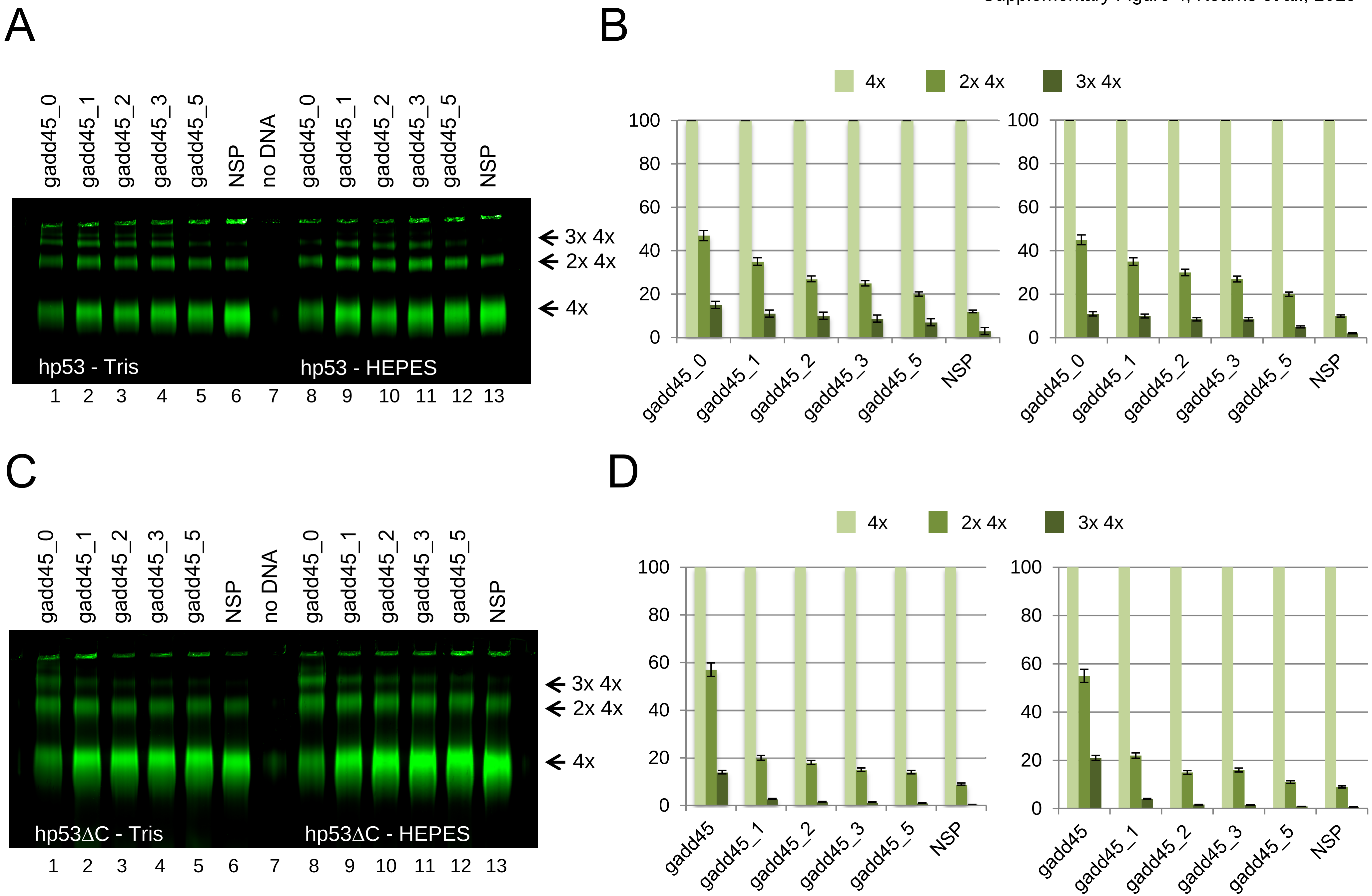
A
gadd45_0
gadd45_1
gadd45_2
gadd45_3
gadd45_5
NSP
no DNA
3x 4x
2x 4x
4x
hp53 - Tris
hp53 - HEPES
1 2 3 4 5 6 7 8 9 10 11 12 13
B
4x
2x 4x
3x 4x
100
80
60
40
20
0
NSP
C
hp53ΔC - Tris
hp53ΔC - HEPES
D
gadd45

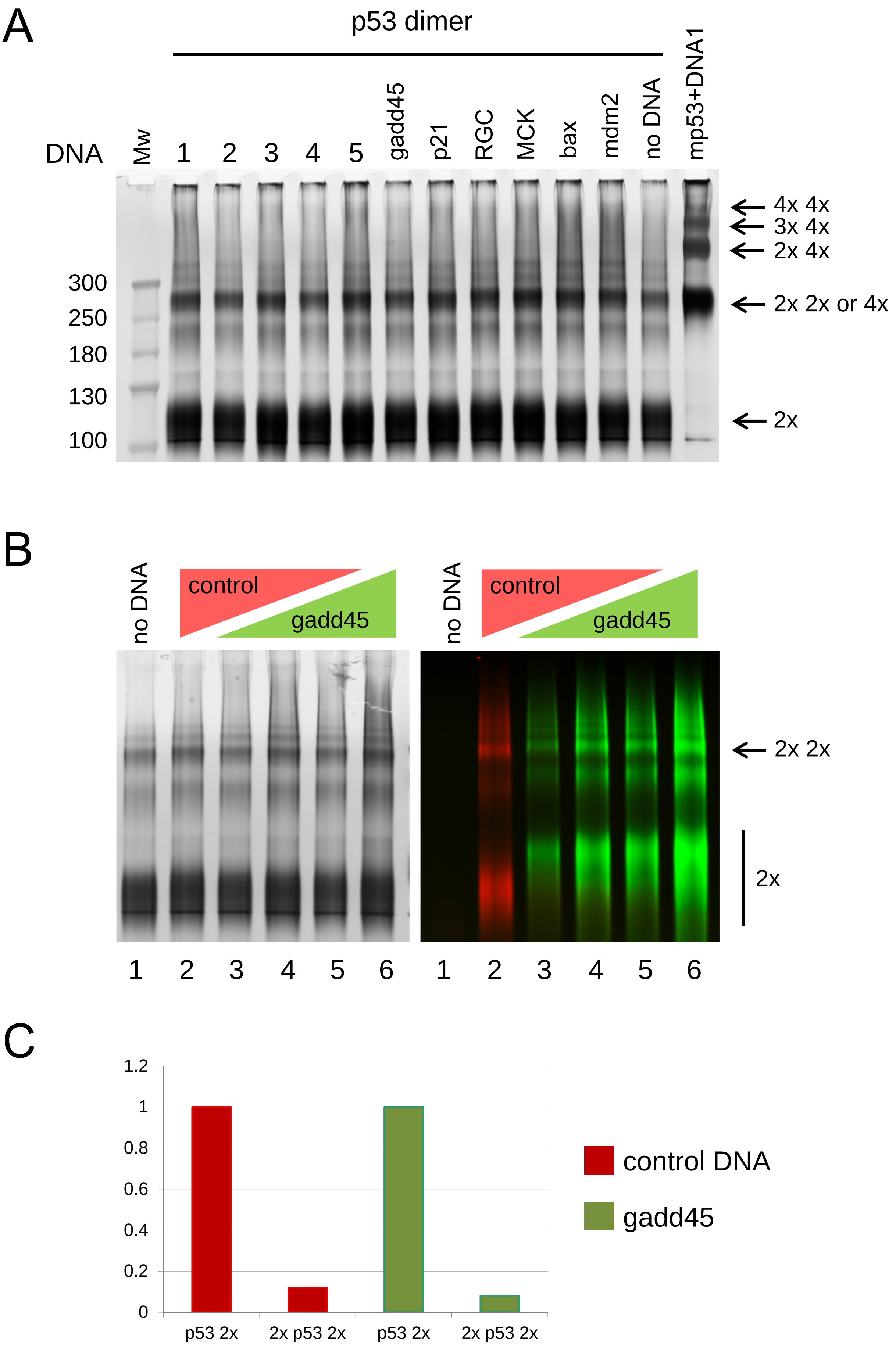
A
p53 dimer
DNA
Mw
1
2
3
4
5
gadd45
p21
RGC
MCK
bax
mdm2
no DNA
mp53+DNA1
300
250
180
130
100
4x 4x
3x 4x
2x 4x
2x 2x or 4x
2x
B
no DNA
control
gadd45
no DNA
control
gadd45
2x 2x
2x
1 2 3 4 5 6 1 2 3 4 5 6
C
1.2
1
0.8
0.6
0.4
0.2
0
p53 2x
2x p53 2x
p53 2x
2x p53 2x
control DNA
gadd45

A

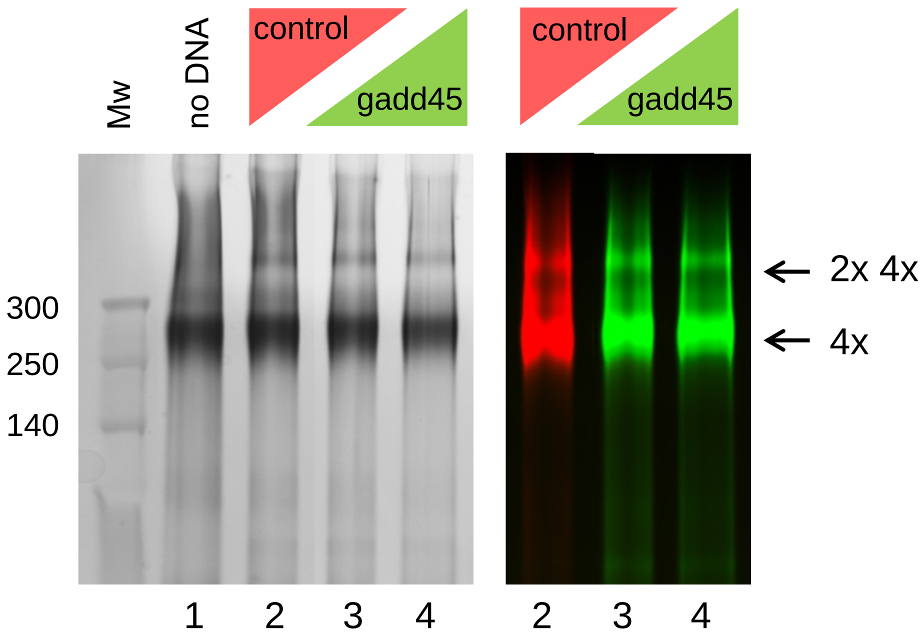
Mw
no DNA
control
gadd45
control
gadd45
300
250
140
2x 4x
4x
1
2
3
4
2
3
4

B

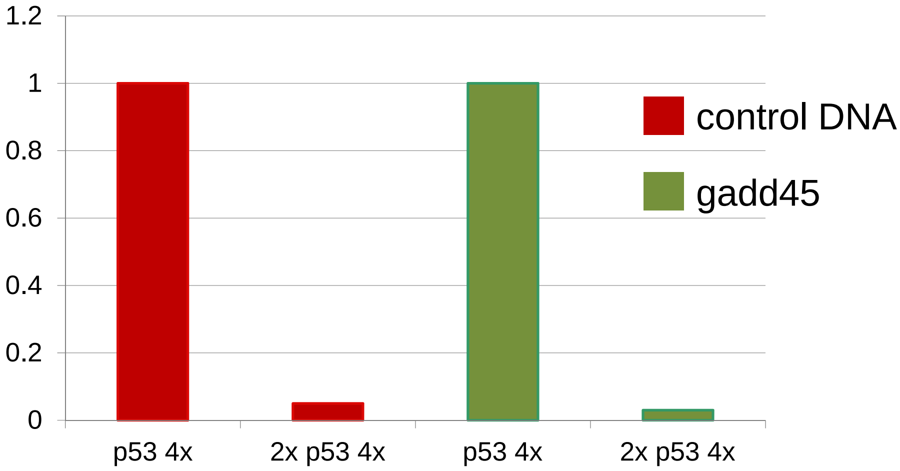
1.2
1
0.8
0.6
0.4
0.2
0
p53 4x
2x p53 4x
p53 4x
2x p53 4x
control DNA
gadd45

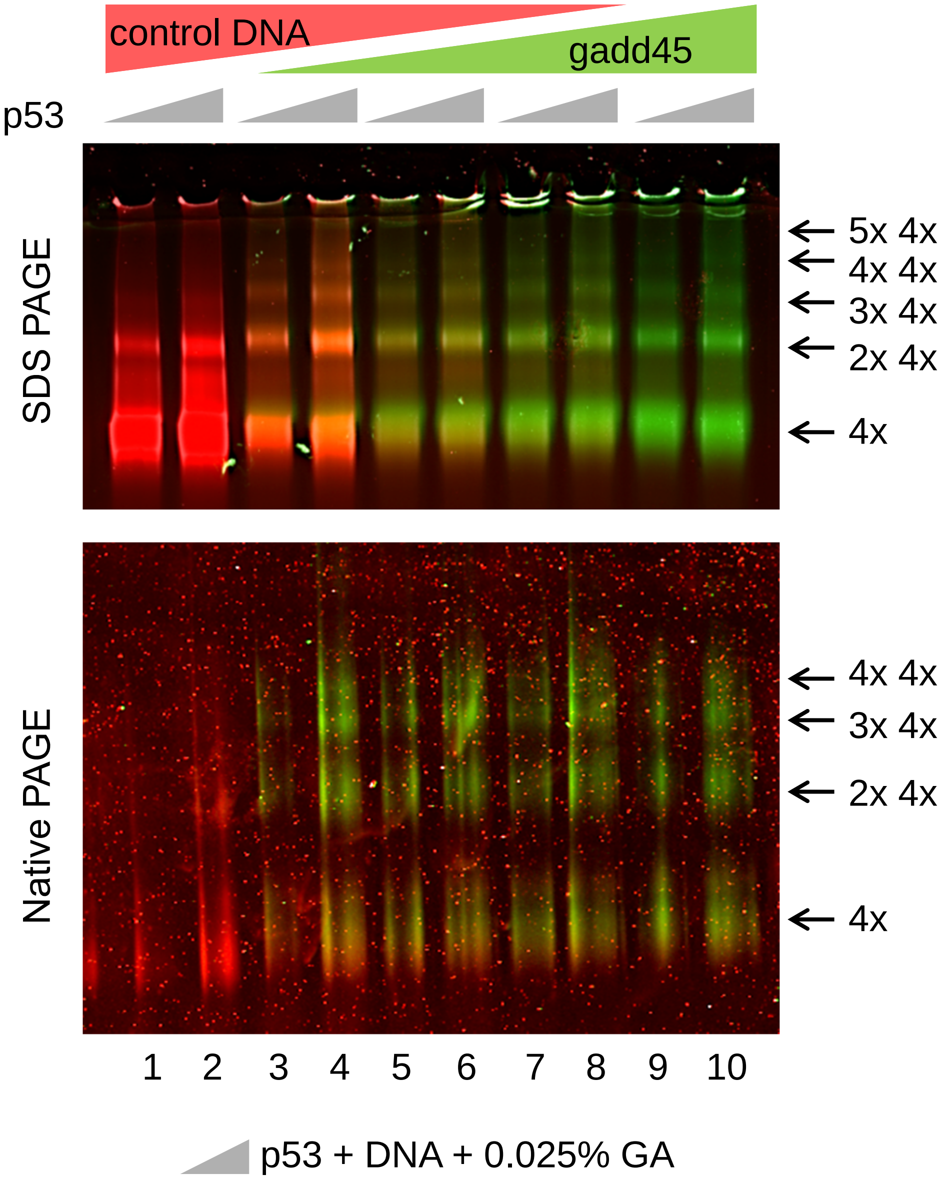
control DNA
gadd45
p53
SDS PAGE
Native PAGE
5x 4x
4x 4x
3x 4x
2x 4x
4x
4x 4x
3x 4x
2x 4x
4x
1 2 3 4 5 6 7 8 9 10
p53 + DNA + 0.025% GA

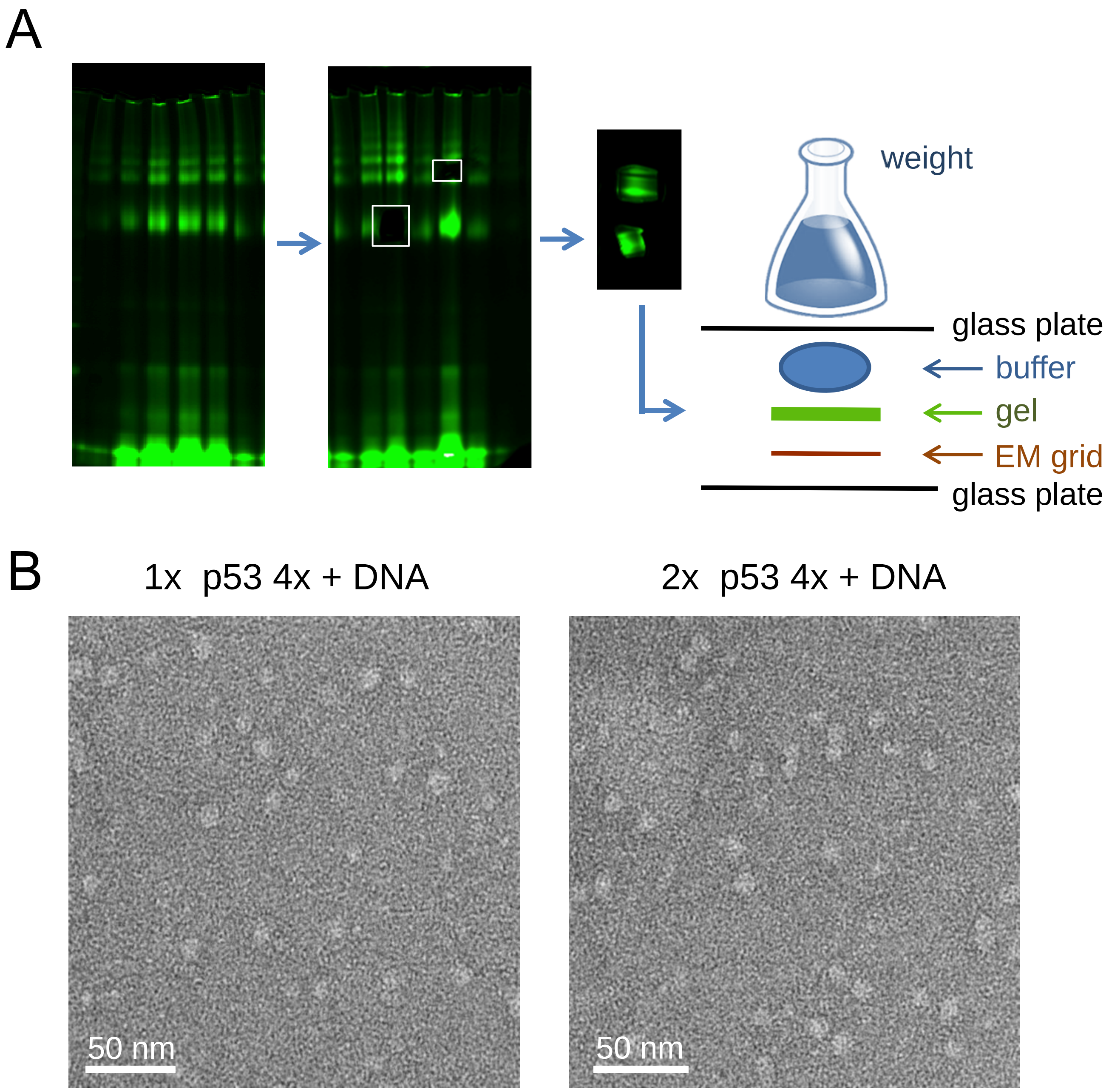
A
weight
glass plate
buffer
gel
EM grid
glass plate
B
1x p53 4x + DNA
2x p53 4x + DNA
50 nm
50 nm

Supplementary Table 1. Oligonucleotide DNA targets used in p53 DNA-binding experiments for Supplementary Figures 2 and 3. Corresponding forward (fwd) and the complementary reverse (rev) oligonucleotides were annealed to each other produce the double-stranded DNA targets.

DNA targets 21-25 were derived from DNA targets 1-5 (Table 1) with addition of extra flanking sequences to increase the overall length of the DNA targets to 66 bp. DNA 20 is fully nonspecific DNA target. All DNA sequences are shown as 5' → 3', spacer and flanking sequences within and up- and down-stream of p53 RE regions are in black, conserved specific sequences within p53 RE regions are in blue, nonspecific sequences within p53 RE regions are in red.

DNA20_FWD agcattggctttctggccatcaggaaaatttcccaaaatttgagctctggcatagaagaggcgct
DNA20_REV agcgcctcttctatgccagagctcaaaattttgggaaattttcctgatggccagaaagccaatgct

DNA21_FWD agcattggctttctggccatcagAGACATGCCTAGACATGCCTagctctggcatagaagaggcgct
DNA21_REV agcgcctcttctatgccagagctAGGCATGTCTAGGCATGTCTctgatggccagaaagccaatgct

DNA22_FWD agcattggctttctggccatcagAGACATGCCTAGACAaattaagctctggcatagaagaggcgct
DNA22_REV agcgcctcttctatgccagagcttaattTGTCTAGGCATGTCTctgatggccagaaagccaatgct

DNA23_FWD agcattggctttctggccatcagAGACATGCCTatgaaTGCCTagctctggcatagaagaggcgct
DNA23_REV agcgcctcttctatgccagagctAGGCAttcatAGGCATGTCTctgatggccagaaagccaatgct

DNA24_FWD agcattggctttctggccatcagatgaaAGACATGCCTttaatagctctggcatagaagaggcgct
DNA24_REV agcgcctcttctatgccagagctattaaAGGCATGTCTttcatctgatggccagaaagccaatgct

DNA25_FWD agcattggctttctggccatcagAGACATGCCTatgaattaatagctctggcatagaagaggcgct
DNA25_REV agcgcctcttctatgccagagctattaattcatAGGCATGTCTctgatggccagaaagccaatgct

DNA31_FWD agcattggctttctggccatcagAGACATGCCTgAGACATGCCTagctctggcatagaagaggcgct
DNA31_REV agcgcctcttctatgccagagctAGGCATGTCTcAGGCATGTCTctgatggccagaaagccaatgct

DNA31_FWD agcattggctttctggccatcagAGACATGCCTgtAGACATGCCTagctctggcatagaagaggcgct
DNA32_REV agcgcctcttctatgccagagctAGGCATGTCTacAGGCATGTCTctgatggccagaaagccaatgct

DNA33_FWD agcattggctttctggccatcagAGACATGCCTgtgAGACATGCCTagctctggcatagaagaggcgct
DNA33_REV agcgcctcttctatgccagagctAGGCATGTCTcacAGGCATGTCTctgatggccagaaagccaatgct

DNA34_FWD agcattggctttctggccatcagAGACATGCCTgtggAGACATGCCTagctctggcatagaagaggcgct
DNA34_REV agcgcctcttctatgccagagctAGGCATGTCTccacAGGCATGTCTctgatggccagaaagccaatgct

DNA35_FWD agcattggctttctggccatcagAAGACATGCCTgtggaAGACATGCCTagctctggcatagaagaggcgct
DNA35_REV agcgcctcttctatgccagagctAGGCATGTCTAtccacAGGCATGTCTctgatggccagaaagccaatgct

DNA36_FWD agcattggctttctggccatcagAGACATGCCTatgaattaatAGACATGCCTagctctggcatagaagaggcgct
DNA36_REV agcgcctcttctatgccagagctAGGCATGTCTattaattcatAGGCATGTCTctgatggccagaaagccaatgct

DNA37_FWD agcattggctttctggccatcagAGACATGCCTatgccttaatatgaaAGACATGCCTagctctggcatagaagaggcgct
DNA37_REV agcgcctcttctatgccagagctAGGCATGTCTttcatattaaggcatAGGCATGTCTctgatggccagaaagccaatgct

hs_gadd45_FWD agcattggctttctggccatcagGAACATGTCTAAGCATGCTgagctctggcatagaagaggcgcT
hs_gadd45_REV agcgcctcttctatgccagagctcAGCATGCTTAGACATGTTCctgatggccagaaagccaatgct

mm_gadd45_FWD agcattggctttctggccatcagGAGCgTGTCTAAGCTgGTggagctctggcatagaagaggcgct
mm_gadd45_REV agcgcctcttctatgccagagctccACCAGCTTAGACAcGCTCctgatggccagaaagccaatgct

Tigar_SP_FWD agcattggctttctggccatcagAGACATGTCCacAGACTTGTCTagctctggcatagaagaggcgct
Tigar_SP_REV agcgcctcttctatgccagagctAGACAAGTCTgtGGACATGTCTctgatggccagaaagccaatgct

Tigar_NS_FWD agcattggctttctggccatcagAGACATGTCCAGACTTGTCTagctctggcatagaagaggcgct
Tigar_NS_REV agcgcctcttctatgccagagctAGACAAGTCTGGACATGTCTctgatggccagaaagccaatgct

Supplementary Table 2. Oligonucleotide DNA targets used in p53 DNA-binding experiments for Figure 5 and Supplementary Figure 5.

Corresponding forward (fwd) and the complementary reverse (rev) oligonucleotides were annealed to each other produce the double-stranded DNA targets.

The overall length of the DNA targets was 34 bp. DNA 20 is fully nonspecific DNA target. All DNA sequences are shown as 5' → 3', spacer and flanking sequences within and up- and down-stream of p53 RE regions are in black, conserved specific sequences within p53 RE regions are in blue, nonspecific sequences within p53 RE regions are in red. All DNA targets were IR800-labeled at 5' end, synthesized at 100nM scale and HPLC purified.

gadd45_0_FWD **IR800-**atgaaatGAACATGTCTAGGCATGCTGaattaat

gadd45_0_REV **IR800-**attaattCAGCATGCCTAGACATGTTCatttcat

gadd45_1_FWD **IR800-**atgaaatGAACATGTCTgAGGCATGCTGaattaa

gadd45_1_REV **IR800-**ttaattCAGCATGCCTcAGACATGTTCatttcat

gadd45_2_FWD **IR800-**tgaaatGAACATGTCTgtAGGCATGCTGaattaa

gadd45_2_REV **IR800-**ttaattCAGCATGCCTacAGACATGTTCatttca

gadd45_3_FWD **IR800-**tgaaatGAACATGTCTgtgAGGCATGCTGaatta

gadd45_3_REV **IR800-**taattCAGCATGCCTcacAGACATGTTCatttca

gadd45_5_FWD **IR800-**gaaatGAACATGTCTgtgtaAGGCATGCTGaatta

gadd45_5_REV **IR800-**taattCAGCATGCCTtacacAGACATGTTCatttc

NSP_FWD **IR800-**gaaattcgatcctctatgaattaataatta

NSP_REV **IR800-**taattattaattcatagaggatcgaatttc
